# *In Situ* Humoral Immunity to Vimentin in HLA-DRB1*03^+^ Patients With Pulmonary Sarcoidosis

**DOI:** 10.3389/fimmu.2018.01516

**Published:** 2018-07-09

**Authors:** Andrew J. Kinloch, Ylva Kaiser, Don Wolfgeher, Junting Ai, Anders Eklund, Marcus R. Clark, Johan Grunewald

**Affiliations:** ^1^Department of Medicine, Section of Rheumatology, Gwen Knapp Center for Lupus and Immunology Research, University of Chicago, Chicago, IL, United States; ^2^Respiratory Medicine Unit, Department of Medicine, Solna and Center for Molecular Medicine, Karolinska Institutet and Karolinska University Hospital Solna, Stockholm, Sweden; ^3^Proteomics Core Laboratory, Cummings Life Science Center, University of Chicago, Chicago, IL, United States

**Keywords:** sarcoidosis, bronchoalveolar lavage fluid, autoantibodies, vimentin, Löfgren’s syndrome, HLA-DRB1*03

## Abstract

Vimentin has been implicated in pulmonary sarcoidosis as a T-cell autoantigen, particularly in the context of HLA-DRB1*03, the Vα2.3/Vβ22 T-cell receptor (TCR), and Löfgren’s syndrome. As vimentin is a known antigenic target in B-cell-mediated autoimmunity, we investigated *in situ* humoral anti-vimentin responses in pulmonary sarcoidosis and their relationship with HLA-DRB1*03. Sarcoid and healthy control (HC) lung biopsies were analyzed by multi-color confocal microscopy for B-cells, T-cells, proliferation, and vimentin, and compared to tonsillectomy tissue. Bronchoalveolar lavage fluid (BALF) and serum from 48 sarcoidosis patients and 15 healthy volunteers were typed for HLA-DRB1*03 and titrated for antibodies to full-length vimentin, vimentin truncations, and total IgG and IgA by ELISA. Presence of extracellular vimentin in BALF was determined by mass spectrometry and T-cell populations measured by flow cytometry. Sarcoid lung samples, especially from HLA-DRB1*03^+^ patients, contained vimentin-rich tertiary lymphoid structures and corresponding BALF was highly enriched for both IgG and IgA anti-vimentin antibody (AVA) titers. Furthermore, sarcoidosis patient BALF AVA concentrations (expressed as arbitrary units per milligram of total immunoglobulin isotype) correlated with the percentage of CD4^+^ T-cells expressing the Vα2.3/Vβ22 TCR. BALF antibody reactivity to the vimentin N-terminus was most prominent in HCs, whereas reactivity to the C-terminus (Vim_C-term_) was enriched in the sarcoid lung. Specifically, HLA-DRB1*03^+^ patient BALF contained higher concentrations of anti-Vim_C-term_ antibodies than BALF from both HCs and HLA-DRB1*03^−^ patients. Consistent with the lung as a site of AVA production, the concentration of AVAs in BALF was dramatically higher than in matched serum samples. Overall, there was a poor correlation between BALF and serum AVA concentrations. Together, these studies reveal the presence of linked *in situ* recognition of vimentin by both T- and B-cells in HLA-DRB1*03^+^ sarcoidosis patients, associated with a selective humoral immune response to the vimentin C-terminus.

## Introduction

Sarcoidosis is a multisystem granulomatous disorder of unknown etiology that primarily affects the lungs (1). The disease is associated with accumulation of CD4^+^ T-cells in bronchoalveolar lavage fluid (BALF), which is particularly pronounced in patients with the acute disease form, "Löfgren’s syndrome" (LS). LS is distinguished by specific clinical symptoms, including an acute disease onset, bilateral hilar lymphadenopathy (BHL), erythema nodosum (EN), and/or bilateral ankle arthritis, and usually a self-limiting disease course. In patients positive for the HLA-DRB1*03 allele (2), dominance of T-cells, in the BALF (but not in matched peripheral blood), expressing T-cell receptor (TCR) segments Vα2.3 and Vβ22 is indicative of restricted antigen recognition in the inflamed lung. Importantly, a higher frequency of these T-cells associates with more rapid clinical recovery (3–5). Interestingly, in patients with expanded Vα2.3^+^Vβ22^+^ T-cells in the lung, peripheral frequencies remain low. This observation suggests that peripheral blood is a poor surrogate for pathogenic processes in the sarcoid lung.

Although primarily considered a T-cell-driven disorder, evidence for B-cell involvement in sarcoidosis, and the interplay between the two cell types, has been suggested by a direct correlation between the percentage of T-cells and antibody-secreting cells in BALF. Systemic sarcoidosis is associated with polyclonal hypergammaglobulinemia, and frequencies of IgA-, IgG-, and IgM-secreting cells in BALF are found to be proportional to those isolated from matched lung tissue samples (6). Furthermore, single-color immunohistochemistry suggests CD20^+^ B-cells, and to a lesser degree CD138^+^ plasma cells, to be common in sarcoid granulomas (7). Significantly, the correlation of BALF-derived antibody-secreting cells with relative frequencies of B-cells and plasma cells in corresponding lung biopsies (6) suggests BALF to be a good surrogate for immune processes in the lung parenchyma.

Interestingly, EN is considered to result from deposition of immune complexes (8, 9), which suggests a direct role for B-cells and antibody production in LS. However, the search for antigens targeted by humoral immunity in sarcoidosis has thus far been inconclusive. In one protein array screen, IgG antibodies to zinc-finger protein 688 and mitochondrial protein L43 were more abundant in sarcoid BALF than controls and higher in non-LS than LS BALF (10). However, no association with other clinical parameters could be identified. One of the limitations of this study was the lack of normalization of titers to these candidate autoantigens for BALF total immunoglobulin isotype. Therefore, no specific enrichment for antibodies reactive with these antigens could be identified. Furthermore, it is also not known whether these antigens are accessible in sarcoid tissue, or if they, or cross-reactive antigens, drive *in situ* adaptive immunity.

Using mass spectrometric characterization of peptides eluted from sarcoid BALF antigen-presenting cell HLA-DR molecules (11, 12), we have previously identified the type III intermediate filament vimentin as a candidate antigen for driving expansion of Vα2.3^+^Vβ22^+^-expressing CD4^+^ T-cell clones. The C-terminal eluted peptide, DSLPLVDTHSKRTLL, has been shown to trigger IFNγ responses in T-cells from HLA-DRB1*03^+^ patients with active disease (13), and by molecular modeling, ideally fits the peptide-binding cleft of the HLA-DRB1*03-TCR Vα2.3/Vβ22 complex (14). A separate study has also identified vimentin as a component of the Kveim reagent, previously used for diagnostic purposes due to its ability to specifically induce granulomatous responses in sarcoidosis patients (15). Importantly, Kveim-derived vimentin promotes T-cell IFNγ production (16). Vimentin is, therefore, one of the most promising candidates for driving *in situ* expansion of Vα2.3^+^Vβ22^+^ CD4^+^ T-cells in patients expressing HLA-DRB1*03.

Humoral immunity to vimentin has been identified in other inflammatory lesions (17, 18). Most notably, lupus tubulointerstitial nephritis is associated with a dominant *in situ* humoral immune response to vimentin, which is itself highly upregulated in inflamed tissue. Also, higher serum anti-vimentin antibody (AVA) titers correlate with severity of tubulointerstitial inflammation (17). Interestingly, HLA-DRB1*03 carriage is frequent in both systemic lupus erythematosus (more than 60%) (19) and sarcoidosis (30–40% of all patients; ~70% of LS patients) (20). These data suggest that *in situ* adaptive immunity to vimentin might be common in chronic inflammation, especially in patients positive for HLA-DRB1*03.

Given the existing molecular data linking HLA-DRB1*03 with Vα2.3^+^Vβ22^+^ CD4^+^ T-cells, adaptive immunity to vimentin and LS, we in this study focused on the influence of HLA-DRB1*03 carriage on the *in situ* humoral response to vimentin. Having established the presence of structures capable of supporting a T-cell-dependent B-cell response to vimentin, we further investigated the associations between the magnitude and subtypes of AVAs with HLA-DRB1*03 carriage and the extent of the Vα2.3^+^Vβ22^+^ CD4^+^ T-cell response in the inflamed lung. We hereby demonstrate that there is indeed a strong enrichment of BALF C-terminal AVAs in HLA-DRB1*03^+^ sarcoidosis patients. These data infer that in sarcoidosis, HLA-DRB1*03 carriage is associated, *in situ*, with both increased frequencies of Vα2.3^+^Vβ22^+^ CD4^+^ T-cells that can recognize a C-terminal vimentin peptide, and humoral immunity to the vimentin C-terminus. Interestingly, secretion of AVAs in the lung did not correlate with serum AVA titers. Together, these data suggest a self-contained *in situ* adaptive immune response to the vimentin C-terminus in sarcoidosis patients, which is particularly pronounced in those expressing HLA-DRB1*03.

## Materials and Methods

### Study Subjects, Bronchoscopy, and BAL

Bronchoscopy with Bronchoalveolar lavage (BAL) was performed as previously described (21). Forty-eight newly diagnosed sarcoidosis patients (16 females) with a median age of 39.5 years (Table 1) were included in the study. All patients were HLA-typed and diagnosed with sarcoidosis according to criteria established by the World Association of Sarcoidosis and Other Granulomatous Disorders (WASOG) (22). Specifically, these included typical clinical and radiographic manifestations, findings at bronchoscopy with BAL including an elevated CD4/CD8 ratio and, if required, positive biopsies, as well as exclusion of other diagnoses. Twenty-six of the 48 patients were diagnosed with LS, defined as an acute onset, usually with fever, chest radiographic findings of BHL, sometimes with pulmonary infiltrates, and EN and/or bilateral ankle arthritis. At the time of BAL, two patients (one LS and one non-LS) were treated with methotrexate and prednisolone. In addition, one LS patient and one non-LS patient had previously received methotrexate and prednisolone but were untreated at the time of BAL. All other patients included in the study were untreated. In addition, BALF, serum, and BALF cells from 15 healthy volunteers (7 females) with normal chest radiography and matched for frequency of HLA-DRB1*03, were analyzed for comparison.

**Table 1:** Clinical characteristics of sarcoidosis patients and controls.

	Among all sarcoidosis patients (*n* = 48)	Among Löfgren’s syndrome (LS) patients (*n* = 26)	Among non-LS patients (*n* = 22)	Among healthy controls (*n* = 15)
Sex (male/female)	32/16	17/9	15/7	8/7
Age, years	39.5 (33.0–51.0)	36.0 (32.3–42.8)	47.5 (34.0–62.0)	25.0 (21.5–28.5)
Chest radiographic stage 0/I/II/III/IV^a,#^	0/23/21/0/2	0/19/7/0/0	0/4/14/0/2	N/A
Smoking status (never-smoker/former/current)	24/17/7	12/7/7	12/10/0	12/0/3
VC (% of predicted)	91.0 (79.0–98.8)	94.5 (81.5–102.0)	84.5 (76.5–94.5)	N/A
DLCO (% of predicted)	87.0 (77.5–95.0)	88.5 (83.0–101.0)	84.0 (77.0–93.0)	N/A
FEV1 (% of predicted)	85.0 (72.0–93.0)	91.0 (83.0–99.5)	78.5 (66.5–91.3)	105.0 (101.3–108.8)
Bronchoalveolar lavage fluid (BALF) cell concentration (10^6^ cells/L)^¤^	210.7 (123.8–338.3)	218.6 (143.7–342.2)	205.5 (117.3–324.1)	74.7 (44.8–115.4)
% BALF recovery	62.5 (51.5–71.0)	68.0 (58.0–71.8)	57.5 (48.5–63.8)	68.0 (58.0–70.0)
% BALF macrophages	76.9 (60.6–85.6)	81.5 (62.2–88.3)	72.9 (55.2–81.1)	90.0 (87.5–94.4)
% BALF lymphocytes^†^	20.2 (12.4–37.4)	17.5 (10.2–35.7)	21.3 (16.9–41.7)	7.7 (5.0–11.5)
% BALF neutrophils	1.35 (0.6–2.1)	1.7 (0.6–2.2)	1.2 (0.9–1.5)	1.0 (0.4–1.8)
% BALF eosinophils	0.2 (0.0–0.6)	0.2 (0.0–0.6)	0.4 (0.0–0.8)	0.0 (0.0–0.6)
% BALF CD3^+^CD4^+^ cells^‡^	85.9 (80.1–90.8)	89.8 (84.2–92.0)	83.9 (75.8–87.9)	63.5 (57.6–71.5)
BALF CD4/CD8 ratio^φ^	9.1 (6.0–16.9)	14.2 (6.8–18.2)	7.5 (4.7–9.2)	2.1 (1.5–3.2)
HLA-DRB1*03^+^/DRB1*03^−^DRB3*01^+^/DRB1*03^−^DRB3*01^−^*	24/10/14	20/4/2	4/6/12	3/3/9
% Vα2.3^+^Vβ22^+^ CD4^+^ T-cells in BALF^Δ^	4.3 (1.4–7.6)	5.4 (3.2–8.9)	1.1 (0.3–2.8)	0.2 (0.1–0.3)

*^a^Chest radiography staging as follows: stage 0 = normal chest radiography, stage I = enlarged lymph nodes, stage II = enlarged lymph nodes with parenchymal infiltrates, stage III = parenchymal infiltrates without enlarged lymph nodes, and stage IV = signs of pulmonary fibrosis*.

*All percentage values are denoted as median (p25–p75)*.

Comparisons of measures in Table 1 based on HLA-DRB1*03 carriage (by the non-parametric Mann–Whitney *U* test unless otherwise specified) are as follows:

^*^Frequency of LS in sarcoidosis patients: *p* < 0.0001 (Fisher’s exact test) [DRB1*03^+^: 20/24 (83%) > DRB1*03^−^: 6/24 (25%)].

^#^Chest radiographic stage; *p* = 0.0285 (DRB1*03^+^ < DRB1*03^−^).

^¤^BALF cell concentration (10^6^ cells/L); *p* = 0.7657 (DRB1*03^+^ < DRB1*03^−^), *p* < 0.0001 (DRB1*03^+^ > HC), *p* = 0.0006 (DRB1*03^–^ > HC).

^†^% BALF lymphocytes; *p* = 0.0100 (DRB1*03^+^ < DRB1*03^−^), *p* = 0.0031 (DRB1*03^+^ > HC), *p* < 0.0001 (DRB1*03^−^ > HC).

^‡^% BALF CD3^+^CD4^+^ cells; *p* = 0.0469 (DRB1*03^+^ > DRB1*03^−^), *p* = 0.0005 (DRB1*03^+^ > HC), *p* = 0.0025 (DRB1*03^−^ > HC).

^φ^BALF CD4/CD8 ratio; *p* = 0.1281 (DRB1*03^+^ > DRB1*03^−^), *p* < 0.0001 (DRB1*03^+^ > HC and DRB1*03^−^ > HC).

^Δ^% Vα2.3^+^Vβ22^+^ CD4^+^ T-cells in BALF; *p* < 0.0001 (DRB1*03^+^ > DRB1*03^−^), *p* = 0.0068 (DRB1*03^+^ > HC), *p* = 0.1064 (DRB1*03^−^ > HC).

*VC, vital capacity; DLCO, carbon monoxide diffusing capacity; FEV1, forced expiratory volume in 1 s*.

Informed consent was obtained from all subjects and ethical approval granted from the Stockholm County Regional Ethical Committee (approval numbers: 2005/1031-31/2, 2009/20-32, 2011/35-32, and 2012/132-32). Joint usage of samples and data was further authorized by the Institutional Review Board at the University of Chicago (approval numbers: 15065B-AM025 and 15065B-AM030).

### Immunofluorescence Staining of Lung Tissue

Granulomatous paraffin-embedded bronchial mucosal biopsies from four LS and six non-LS patients of varying HLA type, chest radiographic stage, and smoking status were used for detection of CD20, CD3, CD4, ki-67, and vimentin. Mucosal biopsies from three healthy volunteers were included for comparison. Human tonsil tissue served as a positive control. A complete description of the staining procedure and subsequent imaging is provided in Supplementary Material.

### Identification of Extracellular Vimentin by Mass Spectrometry

Presence of free vimentin in BALF samples (previously removed of cells and particulate matter by centrifugation) was determined using pre- or post-concentrated (with 10MWCO columns, AmiconUltra-15, Millipore, Tullagreen, Ireland) BALF, subsequently diluted in reducing Laemmli buffer. BALF was resolved by SDS-PAGE and stained with a Coomassie-based reagent (Instant Blue, Expedeon, Cambridge, UK). Proteins were excised for in-gel trypsinization and mass spectrometry as described previously (17). Comprehensive analyses are specified in Supplementary Material.

### Vimentin Cloning and Protein Purification

Full-length vimentin cloned into the C-terminal 6 His-tag fusion expression vector pET-24b (subsequently used for *E. coli* expression and nickel column purification) was kindly provided by Mor-Vaknin et al. (University of Michigan). Three vimentin truncations were subsequently generated in-house using PCR amplification and cloning back within the BamH1 and Hind111 restriction sites of the multiple cloning site of the pET-24b vector, as detailed in Supplementary Material.

### ELISAs

Anti-vimentin antibody ELISAs were performed as for Kinloch et al. (in preparation). A detailed methodology can be found in Supplementary Material. For each antigen and isotype, a standard curve was generated by performing twofold dilutions of frozen aliquots of standard [consisting of pooled sera containing high titers of antibody to the respective whole antigen or truncation, each standard having a neat concentration of 1,000 arbitrary units (AU)]. AU of antibody titers for each analyte were calculated by interpolation from the standard curve, and subsequently multiplying by its dilution factor. ELISAs were repeated until values could be interpolated within the range of the standard curve. Antibodies titrated to the full-length vimentin molecule (amino acids 1–467) are referred to as AVAs, to the N-terminal truncation (amino acids 1–259) as anti-Vim_N-term long_, to the N-terminal truncation (amino acids 1–137) as anti-Vim_N-term short_, and to the C-terminal truncation (amino acids 260–467) as anti-Vim_C-term_. Neat titers (AU) in the text refer to the interpolated titers multiplied by their respective dilution factors. Concentrations (AU/mg) were calculated by dividing the neat titers (AU) by the analytes’ total immunoglobulin isotype concentration.

Total IgG and IgA concentrations in serum and BALF were titrated using Human IgA and Human IgG ELISA kits (E-80A and E-80G, ICL Inc., Portland, OR, USA).

### Statistical Analysis

Anti-vimentin IgG and IgA titers were interpolated using the hyperbola (X is concentration) non-linear regression function in GraphPad Prism v.7 software (GraphPad Software, Inc., La Jolla, CA, USA). Titers were correlated to clinical parameters and compared between patient groups by one-way analysis of variance. The non-parametric Mann–Whitney *U* test and Wilcoxon’s signed rank test were used for individual comparisons between two groups, and Spearman rank correlation for association between BALF and serum AVA titers, as well as AVA titers and BALF CD4/CD8 ratio, percentage of TCR Vα2.3/Vβ22 CD4^+^ T-cells in BALF, and lung function parameters. *p* < 0.05 was considered significant.

## Results

### Clinical Characteristics and Pulmonary Cell Composition Differ Between HLA-DRB1*03 Positive and Negative Patients and Healthy Controls (HCs)

**Figure 1:**
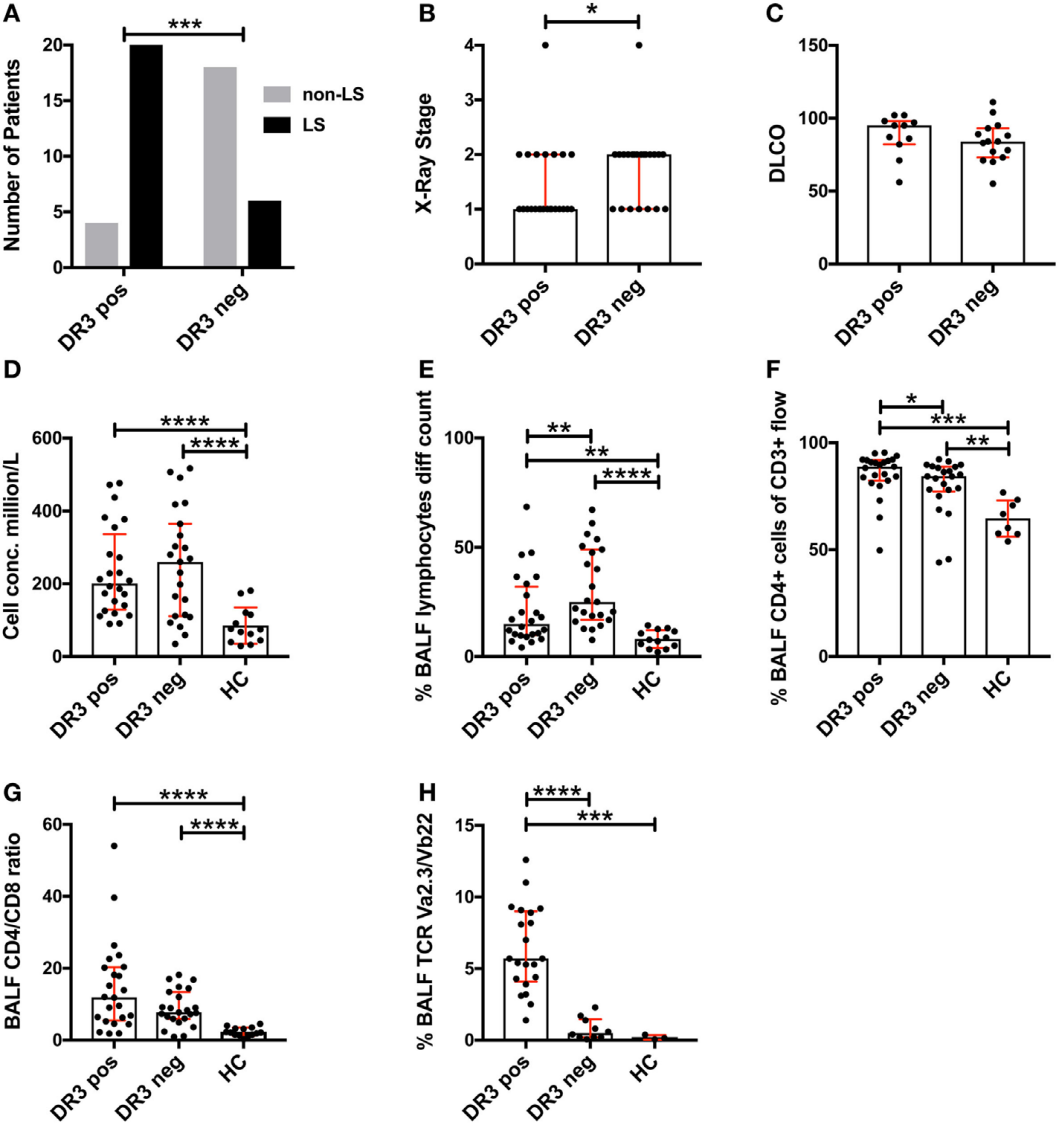
Differences in lung characteristics of HLA-DRB1*03 positive and negative patients and healthy controls. Analysis of clinical characteristics and bronchoalveolar lavage fluid (BALF) parameters of the study cohort was performed according to standard diagnostic routines. Frequency of Löfgren’s syndrome (LS) and non-LS, respectively, in patients positive or negative for HLA-DRB1*03 was determined by Fischer’s exact test **(A)** following HLA typing and independent verification of diagnosis by at least two pulmonary specialists. Similarly, differences in chest radiographic stage **(B)** and DLCO **(C)** were assessed using standard clinical measurements. BALF cell measurements **(D–H)** were performed by *ex vivo* cell count **(D,E)** and flow cytometric analysis **(F–H)** following separation of cells and fluid by centrifugation. Comparisons between groups were performed using the non-parametric Mann–Whitney *U* test and all values are presented as median with interquartile range. Statistically significant differences between groups are indicated as two-tailed *p*-values according to the following scheme: *p < 0.05; **p < 0.01; ***p < 0.001; and ****p < 0.0001.

Analysis of the study cohort (Table 1) demonstrated significant differences between groups in terms of clinical characteristics and diagnostic parameters. Specifically, as has been shown in previous studies (4, 20), the majority of HLA-DRB1*03^+^ patients were diagnosed with LS, while the opposite was true for HLA-DRB1*03^−^ patients (Figure 1A). HLA-DRB1*03^+^ patients also presented with lower chest radiographic staging (Figure 1B), consistent with the diagnosis of LS. No significant difference was observed with regards to diffusing capacity of carbon monoxide (DLCO), although HLA-DRB1*03^−^ patients tended toward a more marked reduction in DLCO (Figure 1C). We next compared BALF cell composition between HLA-DRB1*03 positive and negative patients, as well as HCs. As expected, overall cell concentrations (expressed as million cells per liter fluid) (Figure 1D), as well as the percentage of lymphocytes in BALF (Figure 1E), were significantly elevated in patients compared to controls. Notably, while total cell and lymphocyte counts were higher in HLA-DRB1*03^−^ patients (Figures 1D,E), the percentage of CD4^+^ T-cells was significantly higher in HLA-DRB1*03^+^ patients (Figure 1F), suggesting a more CD4^+^ T-cell-driven immune response in those patients. Consequently, HLA-DRB1*03^+^ patients also displayed the highest CD4/CD8 ratios (Figure 1G). Finally, as previously observed (14), HLA-DRB1*03^+^ patients demonstrated marked predominance of CD4^+^ T-cells carrying the Vα2.3/Vβ22 TCR (Figure 1H).

### Large Vimentin-Rich Cellular Aggregates Containing B-Cells and T-Cells Are Abundant in the Sarcoid Lung

**Figure 2:**
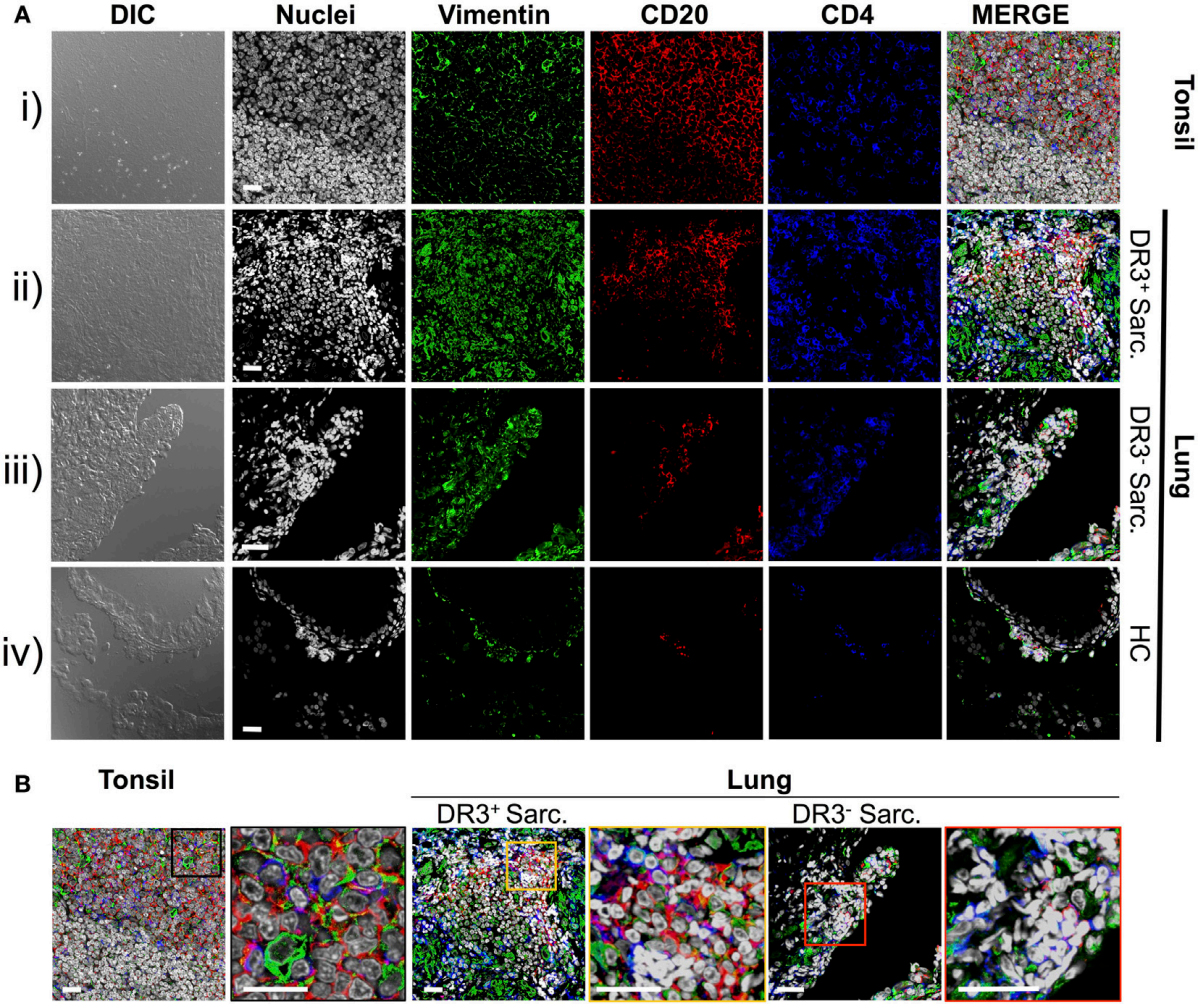
Vimentin, B-cells, and T-cells accumulate and co-localize in sarcoid lung. Specificities of anti-vimentin, anti-CD20 (B-cells), and anti-CD4 (T-cells) antibodies were confirmed by multi-colored immunofluorescence staining of human tonsil [**(A)** i]. Detection was performed using fluorophore-labeled secondary antibodies and false colors subsequently assigned to respective wavelength’s acquired channel. Nuclei were counter-stained with Hoechst. Digital image contrasts of the tissue were also acquired. Analogous stains were performed on HLA-DRB1*03^+^ Löfgren’s syndrome (LS) [**(A)** ii], HLA-DRB1*03^−^ non-LS [**(A)** iii], and healthy [**(A)** iv] mucosal biopsies. Examples of the highest orders of lymphoid organization for each patient group are provided. Magnified areas of merged channels from **(A)** for the respective tissues (as labeled) showing areas positive for vimentin, B-cells, and T-cells are focused on in **(B)**. Negative control images can be found in Supplementary Material. Images were acquired with an oil immersion 40× objective. Scale bar length = 25 µm. Colored boxes correspond to highlighted areas of interest magnified from adjacent images.

Previously, we demonstrated that HLA-DRB1*03^+^ sarcoidosis patients have an *in situ* expansion of Vα2.3^+^Vβ22^+^ T-cells that recognize vimentin peptides in the context of HLA-DRB1*03. We, therefore, sought to determine if this *in situ* T-cell response could be associated with anti-vimentin humoral immunity. As B-cell counts are not routinely assessed in sarcoidosis BALF, we examined B-cell frequency and distribution by multi-color immunofluorescence of mucosal biopsy samples. Therefore, we stained mucosal biopsies or tonsil tissue with antibodies specific for CD20, CD4, and vimentin, and then acquired images using multi-color confocal microscopy (Figure 2).

First, to confirm that we could efficiently stain for the above-mentioned cellular markers using paraffin-embedded tissue, human tonsillectomy tissue was used as a positive control. This tissue type is commonly used in multi-color confocal microscopic analyses of human inflamed lesions as it is readily available, and abundant in lymphocytes and other inflammatory cell types organized in classic secondary lymphoid organ germinal center-type structures. In tonsil samples [Figure 2A(i) and Figure 2B, left panel], T-cell- and B-cell-rich zones suggestive of germinal center light zones were readily apparent. Several cells in these light zone-like structures also expressed vimentin, consistent with the known induction of vimentin in activated T-cells (23) and macrophages (24). In contrast, there was no significant fluorescence in tonsil stained without primary antibodies (Figure S1 in Supplementary Material).

Staining of inflamed sarcoidosis lung samples demonstrated that vimentin was more abundant in the more cellular, inflamed lung than in healthy tissue [Figure 2A(ii–iv) and Figure 2B, middle and right panels]. Vimentin was found in areas containing infiltrating CD4^+^ and CD20^+^ cells. Of the ten (six HLA-DRB1*03^+^ and four HLA-DRB1*03^−^) studied sarcoid biopsies, all but one (from an HLA-DRB1*03^−^ patient) contained large CD4^+^ CD20^+^ vimentin-rich clusters (data not shown). In contrast, in the healthy lung, vimentin expression was primarily restricted to the pulmonary epithelium. Diffuse CD20^+^ cell scatterings were infrequent in the healthy parenchyma (data not shown), and occasional small aggregates of CD4^+^ and CD20^+^ sub-epithelial lymphocytes were also observed [Figure 2A(iv)].

In three of six HLA-DRB1*03^+^ biopsies, large dense aggregates consisting of B-cells, with CD4^+^ T-cells interspersed and around the edges were apparent [Figure 2A(ii) and Figure 2B, middle panel]. In the most inflamed parenchyma, these areas comprised over half of the biopsy area and contained zones reminiscent of structures observed in tonsils. In two of six HLA-DRB1*03^+^ biopsies, and four out of four HLA-DRB1*03^−^ biopsies, aggregates were smaller and large B-cell populations were less evident.

To further verify the localization of B-cell clusters to the sarcoid granuloma, and to quantify potential differences in B-cell abundance in HLA-DRB1*03 positive and negative patients, additional analyses were performed involving the generation of digitally stitched fields of view, as well as hematoxylin and eosin (H&E) stains of granulomatous structures (Figure S2 in Supplementary Material). By comparison of the area and integrated pixel intensity of the largest single individual CD20^+^ zone from each biopsy, HLA-DRB1*03^+^ patients (Figures S2A–C in Supplementary Material) were shown to comprise significantly larger and denser CD20^+^ zones than HLA-DRB1*03^−^ patients (Figures S2D–F in Supplementary Material), further supporting the intensified B-cell response in this patient group (Figure S2G in Supplementary Material). While B-cell clusters in HLA-DRB1*03^−^ patients were small and primarily located around the granuloma, B-cell zones in HLA-DRB1*03^+^ patients were spread throughout the parenchyma (where only diffuse scatterings were found in the healthy lung), even when granulomatous structures were not evident.

### Tertiary Lymphoid Structures in the Inflamed Lung Harbor ki-67^+^ Cells

**Figure 3:**
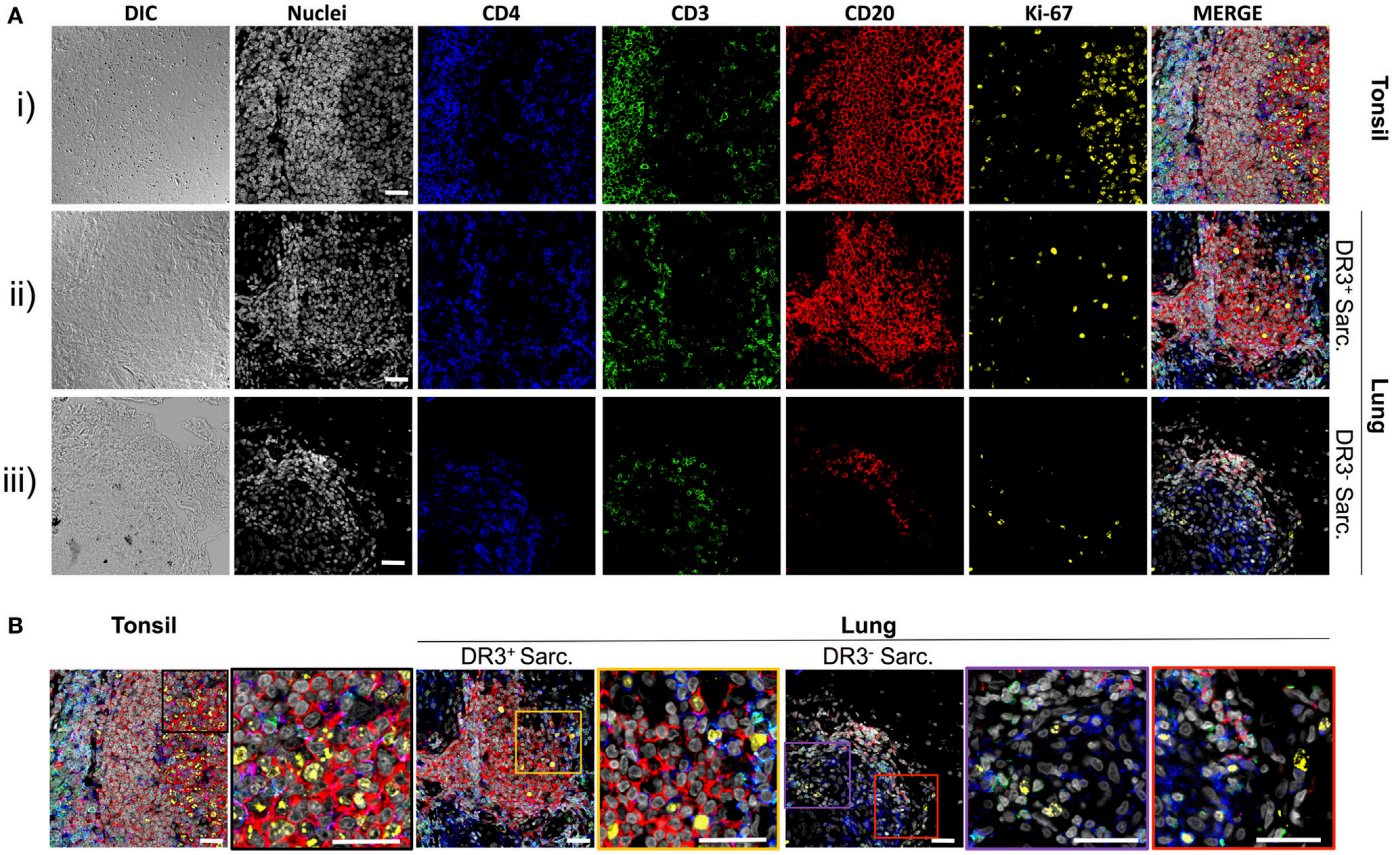
T-cells, B-cells, and proliferating cells in tonsil, HLA-DRB1*03^+^, and HLA-DRB1*03^−^ sarcoid lung. As for Figure 2, tonsil was used as a positive control for multi-color confocal microscopy, but using CD4^+^ T-cell markers CD3 and CD4, the B-cell marker CD20, and the proliferation marker ki-67 [**(A)** i]. Analogous stains were performed on HLA-DRB1*03^+^ LS [**(A)** ii] and HLA-DRB1*03^−^ non-LS lung [**(A)** iii] tissue biopsies. Magnified areas of merged channels labeled showing typical proliferating cell populations are detailed in **(B)**. Images were acquired with an oil immersion 40× objective. Scale bar length = 25 µm. Colored boxes correspond to highlighted areas of interest magnified from adjacent images.

To explore if the sarcoid lung tertiary lymphoid structures could be sites of lymphocyte expansion, tissue samples were stained for ki-67, which marks cells that have recently proliferated, as well as for CD4^+^ T-cells and CD20^+^ B-cells (Figures 3A,B). In tonsils [Figure 3A(i) and Figure 3B, left panel], frequent proliferating CD20^+^ B-cells were observed interspersed with, and surrounded by, CD3^+^CD4^+^ T-cells. Likewise, tissue from HLA-DRB1*03^+^ patients showed a similar pattern of ki-67^+^ cells in and around B-cell: T-cell clusters, with both B-cells and T-cells often expressing ki-67 [Figure 3A(ii) and Figure 3B, middle panel]. The HLA-DRB1*03^−^ aggregates contained fewer B-cells (Figure S2 in Supplementary Material), and CD20^+^ B-cell proliferation was less evident [Figure 3A(iii) and Figure 3B, right panel].

### Total Antibody Responses Are Enriched in the Sarcoid Lung

**Figure 4:**
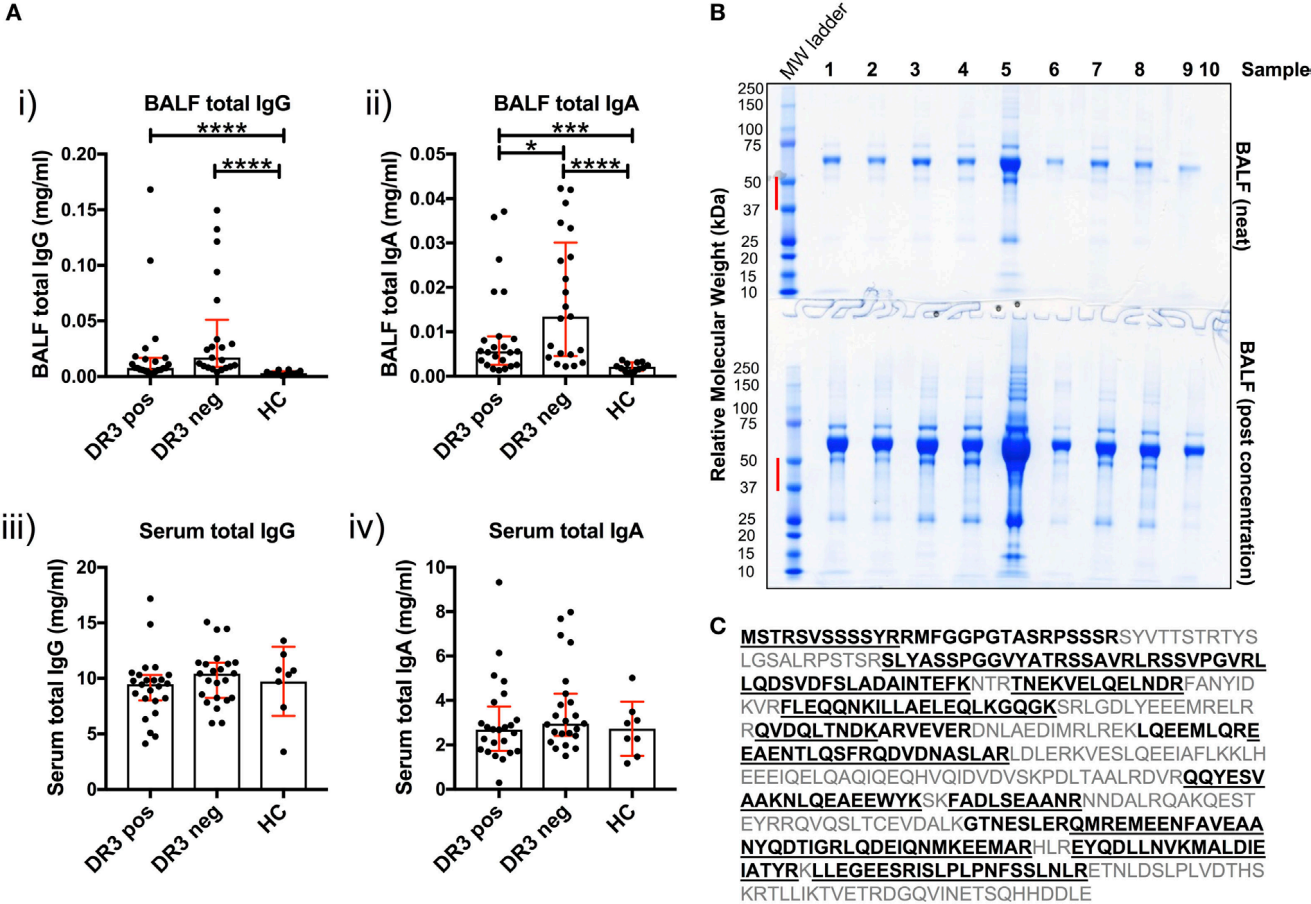
Soluble IgG, IgA, and extracellular vimentin in bronchoalveolar lavage fluid (BALF). The respective total IgG and IgA concentrations of all BALF samples [**(A)** i–ii] were first determined by ELISA, and compared to serum concentrations [**(A)** iii–iv]. Data comparisons performed using the non-parametric Mann–Whitney *U* test. Values are presented as median with interquartile range, *p < 0.05; **p < 0.01; ***p < 0.001; and ****p < 0.0001. Selected samples were subsequently concentrated by size-exclusion chromatography, or left neat and resolved on 4–12% Bis-Tris-gels by reducing SDS-PAGE **(B)**. The molecular weight range (red bar) for each of the lanes of Coomassie-stained gels was excised for each sample. Relative molecular weights as determined by the molecular weight ladder (far left lane) are indicated (kDa). All vimentin peptides determined by mass spectrometry are highlighted in emboldened black, and those confirmed to >97% probability underlined **(C)**. BALF samples run on SDS-PAGE gels were from HLA-DRB1*03^+^ (samples 1–4), HLA-DRB1*03^−^ patients (samples 5–8), and one healthy control (sample 9). Lane 10 served as a negative control (Laemmli buffer alone). Mass spectrometry and protein concentration data for the respective patient samples are provided in the Supplementary Material.

Antibody production in the inflamed sarcoid lung was determined using cell-depleted BALF. Total IgG and total IgA concentrations were elevated in HLA-DRB1*03^+^ and HLA-DRB1*03^−^ sarcoid lung when compared to HCs [Figure 4A(i–ii)]. Between patient groups, HLA-DRB1*03^+^ individuals tended to have lower total IgG and IgA concentrations, but this only reached statistical significance for IgA. Conversely, no differences in the concentration of serum total isotypes were observed between any of the groups [Figure 4A(iii–iv)].

To determine whether soluble vimentin was present and, therefore, antigenically accessible to B-cells, we assessed SDS-PAGE-resolved cell-free BALF samples by mass spectrometry for vimentin. Due to the wide range of protein concentrations in BALF samples (13–885 µg/ml, see Supplementary Material), samples were also analyzed following concentration, to increase the probability of detecting vimentin. Molecular weight ranges excised for analysis covered a range within which vimentin forms have previously been demonstrated (Figure 4B) (17). Peptide analyses demonstrated that extracellular vimentin was detectable in three of the four HLA-DRB1*03^+^ LS and three of four HLA-DRB1*03^−^ non-LS samples analyzed (Supplementary Material). Multiple vimentin peptides were observed, which in total covered 58% of the vimentin molecule (Figure 4C). Vimentin was also detected in the one healthy control sample analyzed, demonstrating that extracellular vimentin was not restricted to the sarcoid lung.

### BALF IgG and IgA AVA Titers Are Elevated in Sarcoidosis

**Figure 5:**
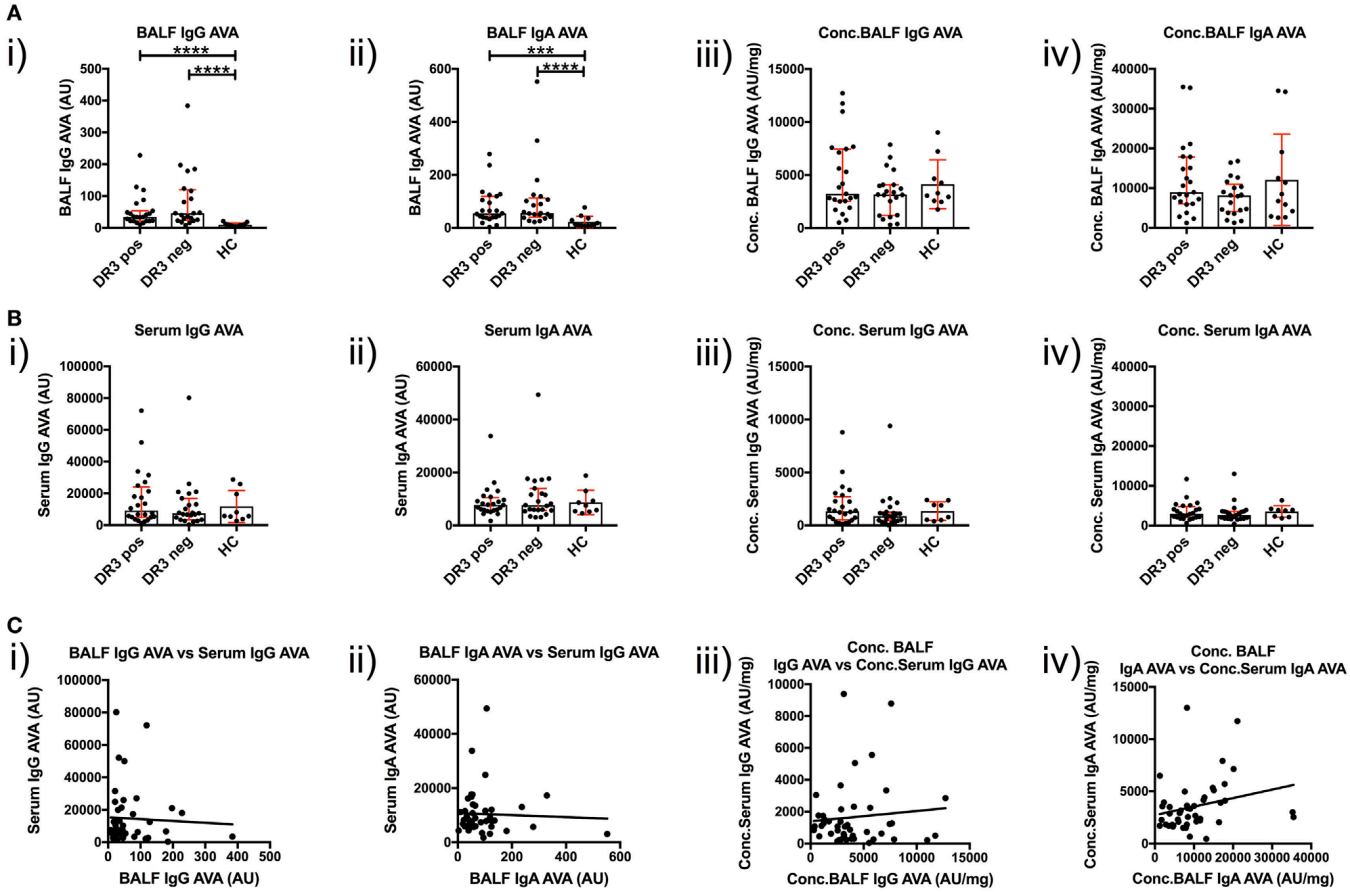
IgG and IgA titers in bronchoalveolar lavage fluid (BALF) and serum to recombinant full-length vimentin. Anti-vimentin antibodies titer differences between sarcoid patients positive and negative for HLA-DRB1*03, and healthy controls (HC), are given for BALF **(A)** and serum **(B)** as neat values [Arbitrary units (AU)] on the left (i,ii), and as concentrations of total immunoglobulin isotype (AU/mg) on the right (iii,iv). Titer comparisons between the two patient groups and HC were performed using the non-parametric Mann–Whitney *U* test and values presented as median with interquartile range. Statistical significances are denoted as two-tailed *p*-values as follows: *p < 0.05; **p < 0.01; ***p < 0.001; and ****p < 0.0001. Spearman correlations of respective antibody titers between matched BALF and serum samples from total sarcoidosis patients (positive and negative for HLA-DRB1*03 combined) are shown in **(C)** for neat values (i,ii) and concentrations (iii,iv) of total immunoglobulin isotype. Lines of best-fit are shown. (i) *r* = −0.020, 95% confidence interval −0.320 to 0.283, *p* = 0.894; (ii) *r* = −0.010, 95% confidence interval −0.310 to 0.293, *p* = 0.950; (iii) *r* = −0.052, 95% confidence interval −0.348 to 0.254, *p* = 0.735; and (iv) *r* = 0.366, 95% confidence interval 0.072 to 0.601, *p* = 0.0135*.

To investigate T-cell-dependent B-cell responses to vimentin in the sarcoid lung, we measured the reactivity of BALF IgG and IgA antibodies with full-length human vimentin using a custom-made ELISA. As demonstrated in Figure 5A(i–ii), neat titers of AVAs were elevated in sarcoidosis patients, but comparable between the HLA-DRB1*03^+^ and HLA-DRB1*03^−^ patient subgroups. In contrast, when neat AVA titers were corrected for concentrations of total IgG and IgA (AU/mg), there were no significant differences in AVA corrected titers between either sarcoidosis patient group or HCs [Figure 5A(iii–iv)].

### AVA Concentrations Are Higher in the Lung Than Peripheral Blood

Anti-vimentin antibodies titers were also measured in matched peripheral blood samples (Figure 5B). No difference was observed in the serum between sarcoidosis and HCs, whether for neat [Figure 5B(i–ii) titers (as AU) or corrected AVA concentrations (as AU/mg), Figure 5B(iii–iv)]. However, it was apparent that corrected AVA concentrations in BALF were much higher than those in serum. For IgG, the concentration of sarcoid BALF AVAs was, on mean average 9.7-fold higher (with a maximum of 150.0-fold) than the corresponding serum concentration, while for IgA AVAs the difference was 4.2-fold (with a maximum of 29.4-fold). AVAs were also elevated in the BALF of HCs for both IgG (average 4.9-fold, maximum 6.5-fold) and IgA (average 5.6-fold, maximum 10.3-fold). These data indicate that, in both HCs and sarcoidosis patients, AVAs are preferentially secreted in the lung. However, in some sarcoid patients, lung AVA secretion is dramatically increased.

We next examined if there was a relationship between BALF and serum AVA concentrations [Figure 5C(i–iv)]. Remarkably, the only association between patient lung and serum AVA levels that reached statistical significance was for concentration of IgA AVA [Figure 5C(iv)]. These data indicate that there is a poor correlation between BALF and serum AVA concentrations.

### Humoral Immunity to the Vimentin C-Terminus in HLA-DRB1*03^+^ Sarcoidosis

**Figure 6:**
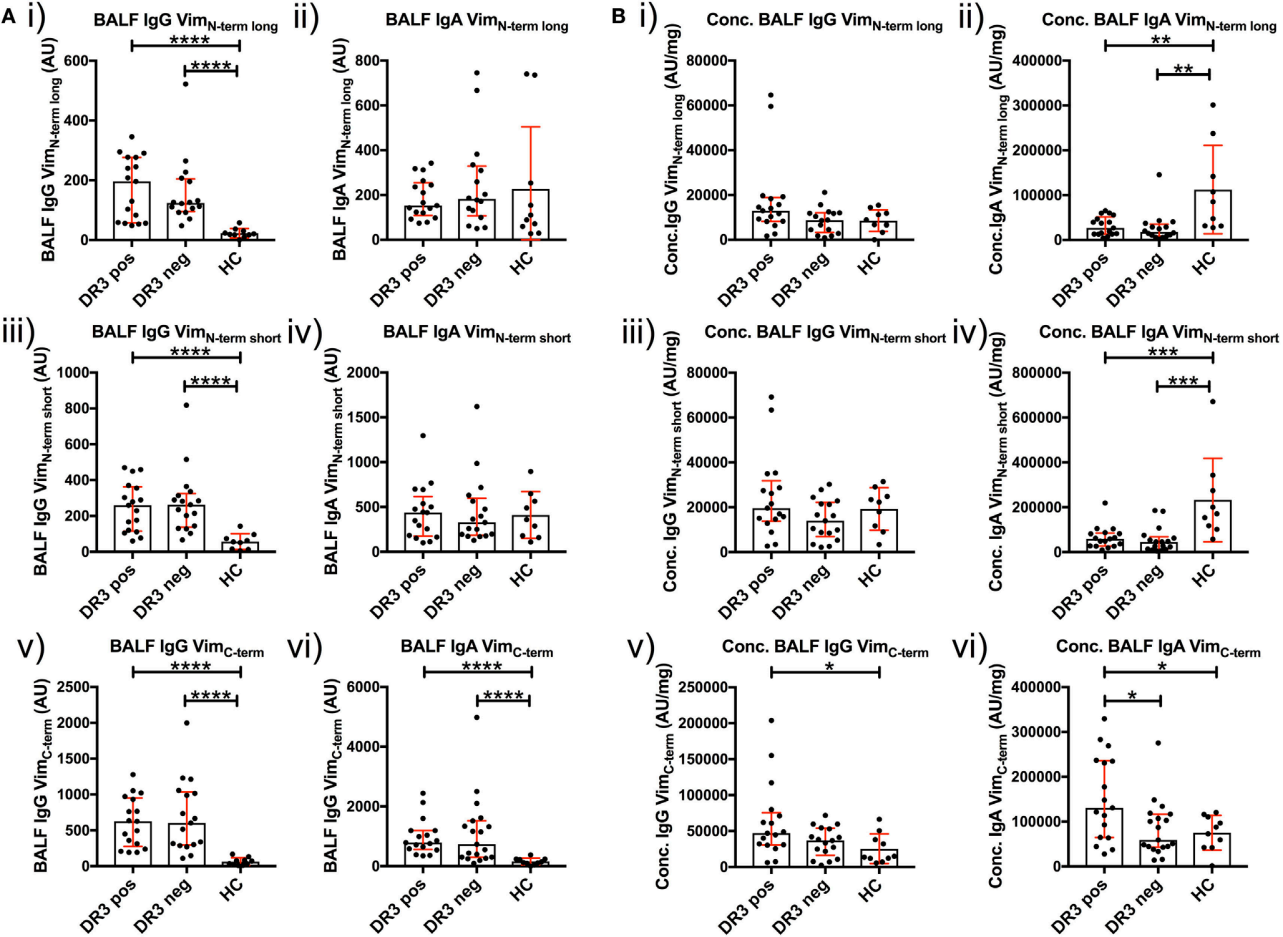
Bronchoalveolar lavage fluid (BALF) IgG and IgA titers to N- and C-terminal vimentin. Differences in BALF titers of IgG and IgA to long (Vim_N-term long_) and short N-terminal (Vim_N-term short_) and a C-terminal recombinant fraction (Vim_C-term_) of vimentin, between sarcoid patient groups and healthy controls are given as neat values [Arbitrary units (AU)] [**(A)** i–vi] and as concentrations of total immunoglobulin isotype (AU/mg) [**(B)** i–vi]. Titer comparisons between the two patient groups and HC were performed using the non-parametric Mann–Whitney *U* test. Values are presented as median with interquartile range and statistical significances are denoted as two-tailed *p*-values as follows: *p < 0.05; **p < 0.01; ***p < 0.001; and ****p < 0.0001.

To determine whether a dominant region of vimentin was selectively targeted by serum and BALF AVAs, we titrated AVA IgG and IgA antibodies to short N-terminal, long N-terminal, and C-terminal vimentin truncations (Figure 6). Neat BALF IgG AVA titers (AU) were higher for each of the three vimentin truncations in sarcoidosis than HCs [Figure 6A(i,iii,v)]. In comparison, for IgA, only neat titers (AU) to the vimentin C-terminus were enriched in sarcoidosis [Figure 6A(ii,iv,vi)]. Interestingly, when comparing BALF titers as concentrations (AU/mg), HCs had higher concentrations of IgA [Figure 6B(ii,iv)], but not IgG [Figure 6B(i,iii)], antibodies to both vimentin N-terminal truncations. In comparison, HLA-DRB1*03^+^ patients demonstrated higher IgA and IgG concentrations to the vimentin C-terminus than HCs and HLA-DRB1*03^−^ patients [Figure 6B(v–vi)]. In contrast, there were no differences in serum AVA concentrations to the different truncations between patient groups and control populations (Figure S3 in Supplementary Material). These data indicate that, in the lung, sarcoidosis patients preferentially secrete AVAs reactive with the C-terminus of vimentin, and that this is augmented with carriage of the HLA-DRB1*03 allele. Conversely, healthy individuals presented with a greater proportion of AVAs specific for the N-terminus. To further emphasize the skewing of the proportion of AVAs targeting the C-terminus in the inflamed lung, the ratio between concentrations of C-terminal and N-terminal (either long or short) AVAs in BALF was calculated for HLA-DRB1*03 positive and negative patients, as well as for HCs (Figures S4 and S5 in Supplementary Material). These analyses confirmed the observation that the proportion of both IgG and IgA antibodies reactive to the C-terminus is elevated in the sarcoid, compared to the healthy lung. Also, comparing these same AVA subtype concentration ratios between BALF and serum within each group, as well as between individual subjects, demonstrated that the proportion of antibodies to the C-terminus was higher in the BALF of sarcoidosis patients (irrespective of HLA-DRB1*03 carriage) but not in HCs, again implicating the sarcoid lung as a site of heightened proportional secretion of antibodies to the vimentin C-terminus.

### AVA Titers Correlate With CD4/CD8 Ratio and Percentage of Vα2.3^+^Vβ22^+^ CD4^+^ T-Cells

**Figure 7:**
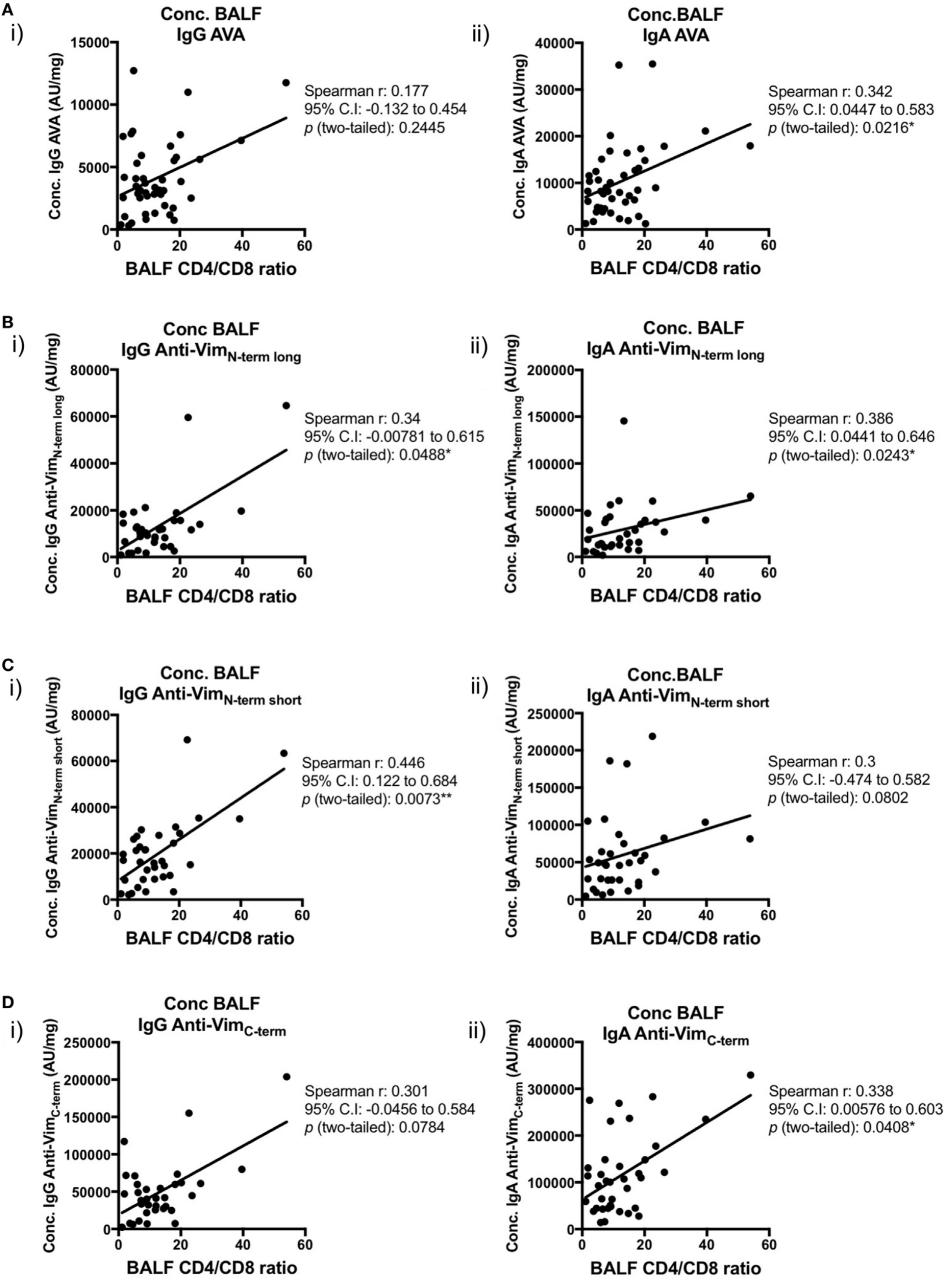
Associations of bronchoalveolar lavage fluid (BALF) CD4/CD8 ratio with BALF antibodies to different vimentin truncations in sarcoidosis patients. Spearman’s correlations between (combined HLA-DRB1*03^+^ and HLA-DRB1*03^−^) patient-matched BALF CD4/CD8 ratios (as determined by flow cytometry) and BALF antibody concentrations (Arbitrary units/mg) to full-length (anti-vimentin antibodies) **(A)** and truncated vimentin **(B–D)** for both IgG (i) and IgA (ii). Lines of best-fit are shown.

**Figure 8:**
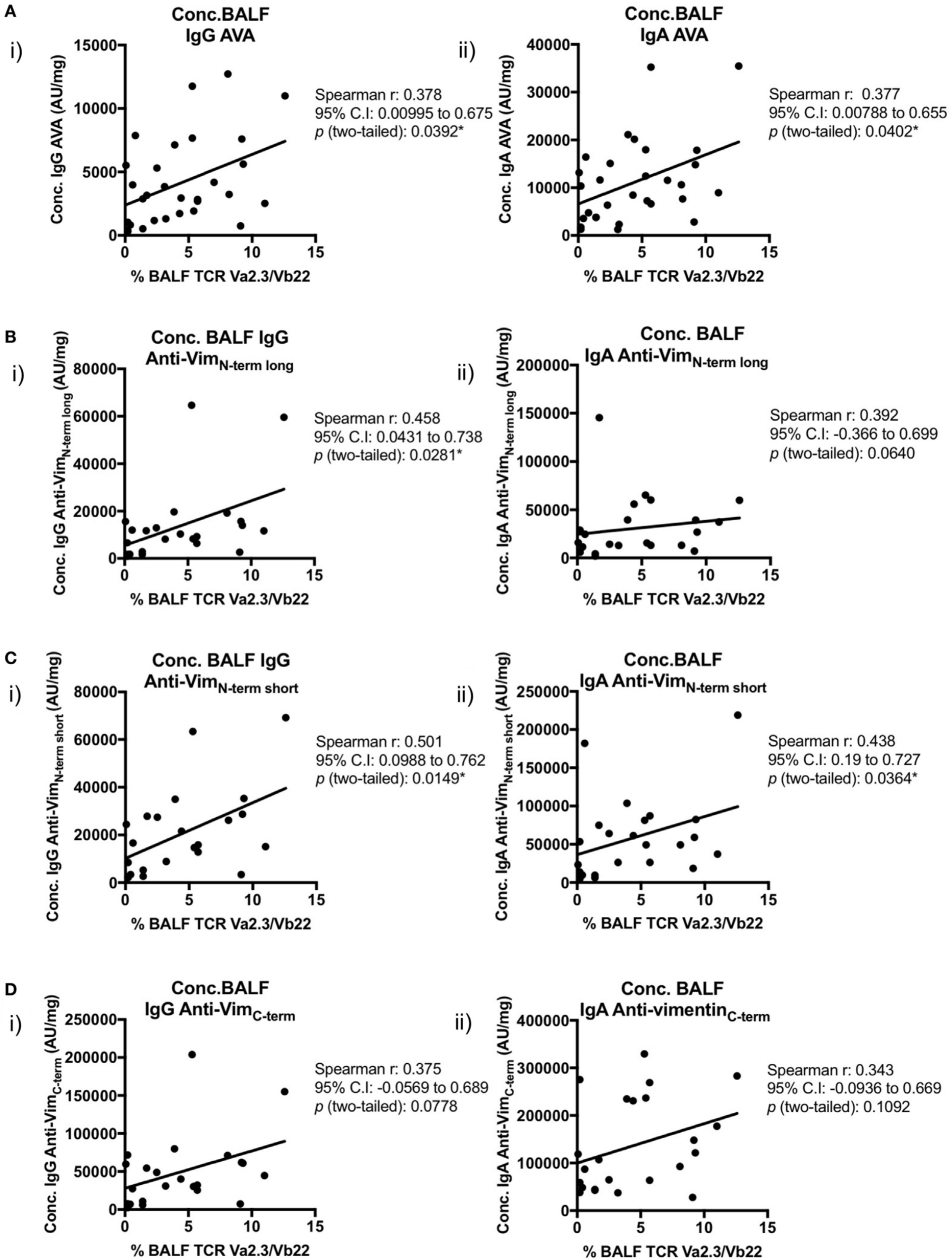
Association of Vα2.3^+^Vβ22^+^ CD4^+^ T-cells in bronchoalveolar lavage fluid (BALF) with antibodies to different vimentin truncations. Spearman’s correlations between (combined HLA-DRB1*03^+^ and HLA-DRB1*03^−^) patient-matched BALF Vα2.3^+^Vβ22^+^ CD4^+^ T-cell percentage (of total CD4^+^ T-cells; as determined by flow cytometry) and BALF antibody concentrations (Arbitrary units/mg) to full-length (anti-vimentin antibodies) **(A)** and truncated vimentin **(B–D)** for both IgG (i) and IgA (ii). Lines of best-fit are shown.

We next examined the relationships between concentrations (AU/mg) of lung-secreted AVAs and BALF T-cell populations (as measured by flow cytometry). For these analyses, all clinical subgroups of sarcoidosis were combined to increase statistical power (Figures 7 and 8). As demonstrated in Figure 7, there was a positive correlation between all assayed IgG and IgA AVA subtype concentrations and CD4/CD8 ratios. For full-length vimentin [Figure 7A(i–ii)], a strong association with IgA AVA concentration was observed, while the association with IgG AVA concentration did not reach statistical significance. There was also a positive correlation between CD4/CD8 ratio and IgA concentrations to both the long N-terminal [Figure 7B(ii)] and C-terminal [Figure 7D(ii)] truncations. In contrast, only concentrations of IgG to the N-terminal domains showed statistically significant positive correlations with CD4/CD8 ratio [Figure 7B(i)] and [Figure 7C(i)].

Importantly, positive correlations were also observed between BALF IgG and IgA AVA subtype concentrations and the percentage of BALF CD4^+^ T-cells expressing the Vα2.3/Vβ22 TCR (Figure 8). Concentrations of both IgA and IgG reactive with full-length vimentin correlated with the proportion of Vα2.3^+^Vβ22^+^ T-cells [Figure 8A(i–ii)], as did both IgG [Figure 8B(i) and Figure 8C(i)] and IgA [Figure 8C(ii)] AVAs to the N-terminal domain. Interestingly, statistical significance was not quite reached in the correlations of Vα2.3/Vβ22 frequency and concentrations of IgG and IgA AVAs to the C-terminus (Figure 8D). However, considering all AVA subtypes collectively, these data strongly suggest that secreted AVA in the lung is associated with clonal expansion of T-cells expressing the Vα2.3/Vβ22 TCR.

### BALF AVA Concentrations Negatively Associate With Lung Function

To determine if AVAs could relate to disease severity, BALF concentrations were compared to DLCO, a measure of lung function (Figure S6 in Supplementary Material). Low DLCOs tended to broadly correlate with high AVA titers. However, only IgA AVA titers against the long N-terminal truncation were statistically associated with low DLCO values (Figure S6B(ii) in Supplementary Material). Nevertheless, these data suggest that high titers of AVAs might be associated with more severe disease.

## Discussion

Primarily described as a T-cell-mediated inflammatory disorder, conclusive evidence of antigen-specific humoral responses in sarcoidosis has been lacking. Furthermore, it was formerly unknown whether T- and B-cells collaborate *in situ* and if this contributes to disease presentation and outcome. In this study, we demonstrate that sarcoidosis can be associated with features of tertiary lymphoid neogenesis, including organized clusters of B- and T-cells, reminiscent of germinal centers typically found in secondary lymphoid organs. Furthermore, our findings implicate vimentin as at least one of the antigens driving *in situ* adaptive immunity in sarcoid lungs. The correlation of BALF AVA concentrations with CD4/CD8 ratio and the frequency of clonally expanded Vα2.3^+^Vβ22^+^ CD4^+^ T-cells further support concomitant propagation of antigen-specific B- and T-cell responses in the lung. Strikingly, while total immunoglobulin secretion in the lungs of HLA-DRB1*03^+^ patients tended to be lower than in HLA-DRB1*03^−^ patients, these patients had a higher concentration of AVAs targeting the vimentin C-terminus, consistent with a more antigenically focused antibody response, yet of lower overall magnitude. The amplification of T- and B-cell clusters throughout the parenchyma in the HLA-DRB1*03^+^ sarcoid lung, where granulomas were not always evident, suggests the more numerous CD20^+^ B-cells to predominantly serve an antigen-presenting function in a local transient inflammation within the inducible bronchus-associated lymphoid tissue.

In sarcoidosis, significant differences in AVA production were observed between HLA-DRB1*03 positive and negative patients and not between LS and non-LS sarcoidosis patients (data not shown). Moreover, AVA concentrations correlated with the percentage of Vα2.3^+^Vβ22^+^ CD4^+^ T-cells in BALF. These observations suggest that HLA-DRB1*03, rather than clinical diagnosis, is the dominant factor in sarcoidosis associated with AVA responses. This conclusion is consistent with previous findings showing expansions of Vα2.3^+^Vβ22^+^ CD4^+^ T-cells in the presence of HLA-DRB1*03, irrespective of other features of LS (14), and highlights the necessity of a future classification of patients based on genetic and/or immunological features rather than symptomatic presentation. At present, HLA-DRB1*03^+^ non-LS and HLA-DRB1*03^−^ LS patients constitute a "gray zone" from a diagnostic and prognostic perspective and would benefit from enhanced understanding of the molecular patterns, such as B- and T-cell responses, that drive disease progression.

Importantly, AVA responses are not exclusive to sarcoidosis. AVA concentrations were also enriched in the BALF of healthy individuals compared to serum, suggesting that constitutive secretion of AVAs is a feature of ordinary lung homeostasis. Indeed, immunoreactivity to vimentin is likely to be a common occurrence in the normal immunoglobulin repertoire. In our own studies, immunoreactivity to vimentin is present in up to 5% of mature and anergic B-cells from healthy donors (Kinloch et al., in preparation). Which domains of vimentin these antibodies commonly target is not known. However, the predominance of N-terminal vimentin immunoreactivity in B-cell lymphomas (25), and the frequent presence of N-terminal reactivity in healthy subjects in the current study, suggest that N-terminal immunoreactivity is a common phenomenon in the healthy B-cell repertoire.

In addition, elevated serum AVAs have been reported in another inflammatory lung disease, interstitial pulmonary fibrosis (26). While this study demonstrated a correlation between serum IgG AVA levels and poor prognosis, it did not determine whether the correlation was also true for BALF AVAs, or if identified AVAs targeted specific vimentin domains. Moreover, it could not be elucidated whether elevated serum AVA titers were associated with *in situ* tertiary lymphoid structures or specific HLA allotypes.

In sarcoidosis patients, we identified both an increased AVA response *in situ* and a shift in vimentin epitopes targeted by AVAs to the C-terminal domain. This was true for both IgG and IgA AVAs and, of the three groups studied, it was HLA-DRB1*03^+^ patients whose total lung antibody populations were most dominated by anti-Vim_C-term_ antibodies. Intriguingly, we have previously demonstrated a C-terminal vimentin peptide that ideally fits into the peptide-binding cleft of this HLA molecule (14). These two observations alone suggest a coordinated epitope shift to the vimentin C-terminus for both B- and T-cell *in situ* adaptive immunity especially in HLA-DRB1*03^+^ sarcoidosis patients. However, when correlating the BALF concentrations of various AVA subtypes with proportions of BALF CD4^+^ T-cells expressing the Vα2.3/Vβ22 TCR, anti-Vim_C-term_ antibodies (although positively associative) did not reach statistical significance. Given the nature of T-cell-mediated B-cell responses, B-cells producing antibodies to the vimentin N-terminus could receive help from CD4^+^ T-cells recognizing the C-terminus. Likewise, B-cells producing antibodies to the vimentin C-terminus could receive help from CD4^+^ T-cells recognizing N-terminal epitopes. The positive, and for the most part statistically significant, correlations of all AVA types with Vα2.3^+^Vβ22^+^ T-cell frequency indicates that this is one of the T-cell clonotypes that can activate any AVA expressing B-cell (irrespective of vimentin epitope recognized by surface Ig) to become an AVA-secreting cell.

The reason for the shift of antibody production to the vimentin C-terminus in the inflamed, and, most notably, HLA-DRB1*03^+^ lung, where the greatest enrichment of these antibodies is observed, remains unclear. Candidate factors include greater accessibility of vimentin C-terminal epitopes during inflammation, and cross-reactivity with similar epitopes on other antigens that become more available in an inflammatory environment.

More qualitative analyses and probing of short overlapping vimentin epitopes, representing the whole vimentin protein, may more definitively reveal whether there are epitopes specifically targeted by antibodies from HLA-DRB1*03^+^ patients. The C-terminal domain is the most likely source of candidates. Also, rather than merely studying relative epitope affinities using soluble antibodies in BALF, frequencies of antibody-secreting, as well as non-secreting B-cell clones, should be compared. The generation of recombinant monoclonal antibodies from dominant clones would further determine which epitopes are most frequently targeted *in situ* in HLA-DRB1*03+ patients and the inflammatory or regulatory nature of such reactive B-cells.

HLA-DRB1*03 is a commonly carried allele, and the adaptive immune response to vimentin is likely to influence pathogenesis in HLA-DRB1*03^+^ patients who suffer from other chronic inflammatory conditions characterized by an active *in situ* lymphocytic component. It is reasonable to hypothesize that in such inflamed lesions, the HLA-DRB1*03 molecule maintains its capacity to present vimentin C-terminal peptides and to support *in situ* T-cell-mediated B-cell responses to antigens including vimentin. While this study specifically focused on this phenomenon in pulmonary sarcoidosis, analogous studies are required in order to determine whether this trend holds true for HLA-DRB1*03^+^ patients with other inflammatory diseases. Moreover, it remains to be elucidated if Vα2.3^+^Vβ22^+^ CD4^+^ T-cells are expanded *in situ* in the lesions of additional HLA-DRB1*03-driven inflammatory diseases. One particularly relevant disease to study is rheumatoid arthritis (RA), which similarly to pulmonary sarcoidosis can present with tertiary lymphoid neogenesis. HLA-DRB1*03^+^ RA patients tend to be anti-CCP seronegative and, reminiscent of HLA-DRB1*03^+^ sarcoidosis patients, have a better prognosis (27–29). Importantly, more comprehensive studies of the influence of HLA-DRB1*03 carriage on antibody production and specificity in the healthy lung are also strongly warranted.

Compared to the lung, there was little corresponding enrichment for AVAs in serum. Neither IgG nor IgA for any of the investigated vimentin substrates showed any consistent relationship between the AVA levels in BALF and serum. This observation is complementary to studies demonstrating a much higher diversity of TCR repertoires in peripheral blood compared to the lung compartment (30) and suggests that adaptive immunity in the sarcoid lung is local, restricted, and self-contained.

The higher concentrations of AVAs in the lung than peripheral blood in both sarcoidosis patients and HCs may reflect two phenomena, both of which are related to more frequent/greater history of local antigenic challenge/exposure in the lung and the subsequent production of local AVA-producing cells. The first of these is challenge from micro-organisms that express vimentin, or cross-reactive antigens, entering the respiratory tract. The second is previous bouts of inflammation that have resulted in local accumulation of vimentin (and cross-reactive host antigens), and the lung’s elicitation of targeted B-cell responses to it. Further studies are, however, required to validate each of these two proposed mechanisms.

As demonstrated here in sarcoidosis, and previously in lupus nephritis (17), vimentin expression is strongly upregulated at sites of inflammation. Upon activation, vimentin is released from macrophages (24) and, accordingly, soluble vimentin is detected in patient BALF samples. This suggests that vimentin is readily available for antigen uptake and presentation in the inflamed lung.

Interestingly, we noted that high AVA titers were associated with reduced lung function. This correlation of heightened antibody concentration with decreased lung function was shared by all AVA subtypes studied, but only reached statistical significance for IgA to the N-terminus. The data presented herein do not allow us to draw any direct pathological conclusions regarding AVAs in pulmonary sarcoidosis. It, therefore, remains to be determined if/how AVAs, of different isotypes and specificities, modulate inflammation *in situ*. Future studies of the function of AVAs, possibly in animal models, are required to investigate their pathogenic roles. AVAs might contribute to tissue damage by forming immune complexes in the extracellular matrix of inflamed tissue where vimentin becomes enriched (31). It is also possible that vimentin has an intrinsic innate inflammatory property [as has been described for another cytoskeletal protein, F-actin, *via* DNGR-1 activation (32)]. If this is indeed the case, binding of AVAs to vimentin at different epitopes is likely to modulate the magnitude of local vimentin-induced inflammation differently. The presence of large extracellular molecular complexes containing vimentin would allow vimentin to cross-link B-cell surface receptors due to antigenic polyvalency. Should vimentin be recognized by danger-associated molecular pattern (DAMP) receptors on B-cells, it may act in a manner analogous to nucleic acids, which activate B-cells by binding to DAMP receptors in endocytic pathways (33). Determination of vimentin’s inflammatory properties (and subsequent motif mapping), together with patient-purified AVA studies are, therefore, central to understanding the pathobiology of AVAs.

In addition to antigenic specificity, other factors might determine whether AVAs are pro- or anti-inflammatory. Indeed, we have recently demonstrated that total IgG antibodies secreted in the lung of sarcoid patients are hypoglycosylated compared to healthy individuals. Furthermore, hypoglycosylated IgG antibodies were most prevalent in non-LS patients who have more severe disease (Heyder et al., in press). These data suggest that *in situ* AVAs, and other *in situ* secreted antibodies, might be more inflammatory in patients with chronic sarcoidosis.

## Conclusion

In summary, our results demonstrate sarcoidosis to be associated with an antigen- and HLA-DRB1*03-restricted humoral immune response to vimentin. Preferential secretion of AVAs in the lung, the local spatial organization of T- and B-cells, availability of antigen and association of AVA secretion with both local CD4^+^ T-cell counts and Vα2.3^+^Vβ22^+^ T-cell frequencies all strongly indicate that these AVAs arise from local, cognate T:B collaboration. Importantly, our data suggest that lung-secreted AVAs in sarcoidosis reflect both an amplification of normal homeostatic immunity and a shift in antigenic specificity from the vimentin N-terminus to the C-terminus. The close link between HLA-DRB1*03, LS, and a good prognosis implies that the nature of *in situ* humoral immunity might reflect fundamental differences in pathogenic mechanisms of prognostic importance. Further studies are, therefore, warranted to gain insight into *in situ* differences between B-cells and antibodies associated with resolving and fibrotic disease.

## Ethics Statement

This study was carried out in accordance with the recommendations of Karolinska Institutet, Karolinska University Hospital, and the Stockholm County Regional Ethical Committee with written informed consent from all subjects in accordance with the Declaration of Helsinki. The protocol was approved by the Stockholm County Regional Ethical Committee. Joint usage of samples and data was further authorized by the Institutional Review Board at the University of Chicago.

## Author Contributions

Conception and study design: YK, AK, MC, and JG. Sample collection: YK and AE. Performing experiments: YK (flow cytometry), AK (cloning, protein purification, and resolution, ELISA, confocal microscopy), DW (mass spectrometry), and JA (confocal microscopy). Analysis and interpretation: YK, AK, and DW. Manuscript preparation: YK, AK, DW, MC, and JG. Critical reading and intellectual assessment of manuscript: YK, AK, DW, AE, MC, JA, and JG. All authors read and approved the final manuscript.

## Conflict of Interest Statement

The authors declare that the research was conducted in the absence of any commercial or financial relationships that could be construed as a potential conflict of interest.

## References

[1] Iannuzzi MC Rybicki BA Teirstein AS Sarcoidosis. N Engl J Med (2007) 357(21):2153–65. 10.1056/NEJMra071714 18032765

[2] Berlin M Fogdell-Hahn A Olerup O Eklund A Grunewald J HLA-DR predicts the prognosis in Scandinavian patients with pulmonary sarcoidosis. Am J Respir Crit Care Med (1997) 156(5):1601–5. 10.1164/ajrccm.156.5.9704069 9372682

[3] Grunewald J Berlin M Olerup O Eklund A. Lung T-helper cells expressing T-cell receptor AV2S3 associate with clinical features of pulmonary sarcoidosis. Am J Respir Crit Care Med (2000) 161(3 Pt 1):814–8. 10.1164/ajrccm.161.3.9906001 10712327

[4] Grunewald J Eklund A Lofgren’s syndrome: human leukocyte antigen strongly influences the disease course. Am J Respir Crit Care Med (2009) 179(4):307–12. 10.1164/rccm.200807-1082OC 18996998

[5] Planck A Eklund A Grunewald J. Markers of activity in clinically recovered human leukocyte antigen-DR17-positive sarcoidosis patients. Eur Respir J (2003) 21(1):52–7. 10.1183/09031936.03.00059103 12570109

[6] Hunninghake GW Crystal RG. Mechanisms of hypergammaglobulinemia in pulmonary sarcoidosis. Site of increased antibody production and role of T lymphocytes. J Clin Invest (1981) 67(1):86–92. 10.1172/JCI110036 6969734 PMC371575

[7] Kamphuis LS van Zelm MC Lam KH Rimmelzwaan GF Baarsma GS Dik WA Perigranuloma localization and abnormal maturation of B cells: emerging key players in sarcoidosis? Am J Respir Crit Care Med (2013) 187(4):406–16. 10.1164/rccm.201206-1024OC 23239158

[8] Hedfors E Norberg R Evidence for circulating immune complexes in sarcoidosis. Clin Exp Immunol (1974) 16(3):493–6. 4468851 PMC1553941

[9] Jones JV Cumming RH Asplin CM. Evidence for circulating immune complexes in erythema nodosum and early sarcoidosis. Ann N Y Acad Sci (1976) 278:212–9. 10.1111/j.1749-6632.1976.tb47032.x 1067006

[10] Haggmark A Hamsten C Wiklundh E Lindskog C Mattsson C Andersson E Proteomic profiling reveals autoimmune targets in sarcoidosis. Am J Respir Crit Care Med (2015) 191(5):574–83. 10.1164/rccm.201407-1341OC 25608002

[11] Heyder T Kohler M Tarasova NK Haag S Rutishauser D Rivera NV Approach for identifying human leukocyte antigen (HLA)-DR bound peptides from scarce clinical samples. Mol Cell Proteomics (2016) 15(9):3017–29. 10.1074/mcp.M116.060764 27452731 PMC5013314

[12] Wahlstrom J Dengjel J Persson B Duyar H Rammensee HG Stevanovic S Identification of HLA-DR-bound peptides presented by human bronchoalveolar lavage cells in sarcoidosis. J Clin Invest (2007) 117(11):3576–82. 10.1172/JCI32401 17975675 PMC2045606

[13] Wahlstrom J Dengjel J Winqvist O Targoff I Persson B Duyar H Autoimmune T cell responses to antigenic peptides presented by bronchoalveolar lavage cell HLA-DR molecules in sarcoidosis. Clin Immunol (2009) 133(3):353–63. 10.1016/j.clim.2009.08.008 19786367

[14] Grunewald J Kaiser Y Ostadkarampour M Rivera NV Vezzi F Lotstedt B T-cell receptor-HLA-DRB1 associations suggest specific antigens in pulmonary sarcoidosis. Eur Respir J (2016) 47(3):898–909. 10.1183/13993003.01209-2015 26585430

[15] Kveim A En ny og spesifikk kutan-reaksjon ved Boecks sarcoid [A new and specific cutaneous reaction in Boeck’s sarcoid]. Nord Med (1941) 9:169–72.

[16] Eberhardt C Thillai M Parker R Siddiqui N Potiphar L Goldin R Proteomic analysis of kveim reagent identifies targets of cellular immunity in sarcoidosis. PLoS One (2017) 12(1):e0170285. 10.1371/journal.pone.0170285 28114394 PMC5256960

[17] Kinloch AJ Chang A Ko K Henry Dunand CJ Henderson S Maienschein-Cline M Vimentin is a dominant target of in situ humoral immunity in human lupus tubulointerstitial nephritis. Arthritis Rheumatol (2014) 66(12):3359–70. 10.1002/art.38888 25306868 PMC4264660

[18] Porcheray F DeVito J Helou Y Dargon I Fraser JW Nobecourt P Expansion of polyreactive B cells cross-reactive to HLA and self in the blood of a patient with kidney graft rejection. Am J Transplant (2012) 12(8):2088–97. 10.1111/j.1600-6143.2012.04053.x 22510337 PMC3402627

[19] Graham RR Ortmann W Rodine P Espe K Langefeld C Lange E Specific combinations of HLA-DR2 and DR3 class II haplotypes contribute graded risk for disease susceptibility and autoantibodies in human SLE. Eur J Hum Genet (2007) 15(8):823–30. 10.1038/sj.ejhg.5201827 17406641

[20] Grunewald J Brynedal B Darlington P Nisell M Cederlund K Hillert J Different HLA-DRB1 allele distributions in distinct clinical subgroups of sarcoidosis patients. Respir Res (2010) 11:25. 10.1186/1465-9921-11-25 20187937 PMC2846896

[21] Olsen HH Grunewald J Tornling G Skold CM Eklund A. Bronchoalveolar lavage results are independent of season, age, gender and collection site. PLoS One (2012) 7(8):e43644. 10.1371/journal.pone.0043644 22952729 PMC3432041

[22] Costabel U Hunninghake GW ATS/ERS/WASOG statement on sarcoidosis. Sarcoidosis Statement Committee. American Thoracic Society. European Respiratory Society. World Association for Sarcoidosis and Other Granulomatous Disorders. Eur Respir J (1999) 14(4):735–7. 10.1034/j.1399-3003.1999.14d02.x 10573213

[23] Huet D Bagot M Loyaux D Capdevielle J Conraux L Ferrara P SC5 mAb represents a unique tool for the detection of extracellular vimentin as a specific marker of Sezary cells. J Immunol (2006) 176(1):652–9. 10.4049/jimmunol.176.1.652 16365461

[24] Mor-Vaknin N Punturieri A Sitwala K Markovitz DM. Vimentin is secreted by activated macrophages. Nat Cell Biol (2003) 5(1):59–63. 10.1038/ncb898 12483219

[25] Cha SC Qin H Kannan S Rawal S Watkins LS Baio FE Nonstereotyped lymphoma B cell receptors recognize vimentin as a shared autoantigen. J Immunol (2013) 190(9):4887–98. 10.4049/jimmunol.1300179 23536634 PMC3633696

[26] Li FJ Surolia R Li H Wang Z Kulkarni T Liu G Autoimmunity to vimentin is associated with outcomes of patients with idiopathic pulmonary fibrosis. J Immunol (2017) 199(5):1596–605. 10.4049/jimmunol.1700473 28754682 PMC5563167

[27] Cui J Taylor KE Lee YC Kallberg H Weinblatt ME Coblyn JS The influence of polygenic risk scores on heritability of anti-CCP level in RA. Genes Immun (2014) 15(2):107–14. 10.1038/gene.2013.68 24385024 PMC3948067

[28] Irigoyen P Lee AT Wener MH Li W Kern M Batliwalla F Regulation of anti-cyclic citrullinated peptide antibodies in rheumatoid arthritis: contrasting effects of HLA-DR3 and the shared epitope alleles. Arthritis Rheum (2005) 52(12):3813–8. 10.1002/art.21419 16320316

[29] Verpoort KN van Gaalen FA van der Helm-van Mil AH Schreuder GM Breedveld FC Huizinga TW Association of HLA-DR3 with anti-cyclic citrullinated peptide antibody-negative rheumatoid arthritis. Arthritis Rheum (2005) 52(10):3058–62. 10.1002/art.21302 16200610

[30] Mitchell AM Kaiser Y Falta MT Munson DJ Landry LG Eklund A Shared alphabeta TCR usage in lungs of sarcoidosis patients with Lofgren’s syndrome. J Immunol (2017) 199(7):2279–90. 10.4049/jimmunol.1700570 28827283 PMC5675165

[31] Didangelos A Puglia M Iberl M Sanchez-Bellot C Roschitzki B Bradbury EJ. High-throughput proteomics reveal alarmins as amplifiers of tissue pathology and inflammation after spinal cord injury. Sci Rep (2016) 6:21607. 10.1038/srep21607 26899371 PMC4761922

[32] Ahrens S Zelenay S Sancho D Hanc P Kjaer S Feest C F-actin is an evolutionarily conserved damage-associated molecular pattern recognized by DNGR-1, a receptor for dead cells. Immunity (2012) 36(4):635–45. 10.1016/j.immuni.2012.03.008 22483800

[33] Avalos AM Busconi L Marshak-Rothstein A. Regulation of autoreactive B cell responses to endogenous TLR ligands. Autoimmunity (2010) 43(1):76–83. 10.3109/08916930903374618 20014959 PMC3059585

