# Supplementary material for: In Situ Humoral Immunity to Vimentin in HLA-DRB1*03+ Patients With Pulmonary Sarcoidosis
Source: Front Immunol. 2018 Jul 9;9:1516. doi: 10.3389/fimmu.2018.01516 (PMC6046378; doi:10.3389/fimmu.2018.01516)
Supplement: Supplementary file 1 [file presentation_1.PDF]

SUPPLEMENTARY MATERIAL

In situ humoral immunity to vimentin in HLA-DRB1*03$^+$ patients with pulmonary sarcoidosis

Andrew J. Kinloch[1]*, Ylva Kaiser[2]*[¤], Don Wolfgeher[1], Junting Ai[1], Anders Eklund[2], Marcus R. Clark[1#], and Johan Grunewald[2#]

*These authors contributed equally to the study.

[#]These authors contributed equally to the study.

[¤]**Correspondence**: Ylva Kaiser, PhD; ylva.kaiser@ki.se

Supplementary Material and Methods

HLA typing

Genomic DNA was extracted from whole blood samples of patients and healthy volunteers. HLA-DRB1 and DRB3 alleles were subsequently determined by the PCR-sequence-specific primer (PCR-SSP) technique (Olerup SSP-DR Low Resolution Kit, Saltsjöbaden, Sweden) as previously described (S1).

Serum and BALF isolation

Whole blood was collected from patients and healthy volunteers at the time of BAL into sodium-heparinised tubes. Serum was isolated as part of the clinical routine investigation, immediately frozen and stored at -80°C, along with matching BALF samples that had been separated from cells and precipitous material by repeated centrifugation.

Measurement of TCR expression by flow cytometry

Freshly isolated BALF cells were stained *ex vivo* using the following antibodies: CD3-Pacific Blue, clone UCHT1 (BD Pharmingen, San Diego, CA, USA), CD4-APC-H7, clone SK3 (BD Biosciences, San Jose, CA, USA), Vα2.3-FITC, clone F1 (Thermo Scientific, Rockford, IL, USA) and Vβ22-PE, clone IMMU 546 (Beckman Coulter Immunotech, Marseille, France). Live/Dead Fixable Aqua Dead Cell Stain Kit (Life Technologies, Eugene, OR, USA) was used for assessment of cell viability. Cells were sequentially gated on lymphocytes (based on FSC vs. SSC), single cells (based on FSC-A vs. FSC-H), viable cells (defined as Aqua negatively stained cells), $CD3^+$ and $CD4^+$ cells. The $CD4^+$ gate was set as threshold gate for acquisition, with a minimum of 15,000 events being collected. Flow cytometry was run on a BD FACS Canto II or a Fortessa X-20 (Beckton Dickinson,

San Jose, CA, USA) and results were analysed using FlowJo X (TreeStar, Ashland, OR, USA) software.

Immunofluorescence staining of lung tissue

Granulomatous paraffin-embedded bronchial mucosal biopsies from four LS and six non-LS patients of varying HLA type, chest radiographic stage and smoking status were used for detection of CD20, CD3, CD4, ki-67 and vimentin. Mucosal biopsies from three healthy volunteers were included for comparison. Healthy human tonsil tissue served as a positive control. Tissue on slides was deparaffinised by baking for 30 minutes at 60°C and rehydrated by sequential immersions in xylene (three times ten minutes), 100% ethanol (two times five minute), 95% ethanol (two times five minutes), 70% ethanol (five minutes) and ddH_2O (five minutes). Antigen retrieval was performed by immersion in citrate buffer (DakoS1699, Agilent, Santa Clara, CA, USA) for twenty minutes at 96°C and twenty minutes at room temperature, and washing performed in ddH_2O (five minutes) and TBS (three times five minutes). Tissue was blocked for two hours in blocking buffer (TBS/0.02% Triton X-100/10% normal donkey serum/1:500 Human BD Fc Block [BD Pharmingen, San Diego, CA, USA]) and incubated for two hours with primary antibodies Alexa Fluor 647-rabbit monoclonal anti-CD4 (Abcam ab196147, Cambridge, United Kingdom), monoclonal mouse anti-CD20 (Dako, clone L26, Glostrup, Denmark), and polyclonal goat anti-vimentin (Abcam ab11256) diluted in blocking buffer. Primary antibodies were washed with TBS (three times two minutes) and secondary antibodies (Molecular Probes, Invitrogen, Eugene, OR, USA) Alexa Fluor 488-donkey anti-goat, Alexa Fluor 564-donkey anti-mouse (diluted in blocking buffer/1:500 Hoechst 33342 [Molecular Probes, Invitrogen, Eugene, OR, USA]) added for an additional two hours and washed off before addition of SlowFade Gold Antifade (Molecular Probes, Invitrogen) and cover glass (SLIP-RITE, cat.no 102260, Richard-Allan Scientific,

Kalamazoo, MI, USA). Reactivity of primary antibodies for indicated antigens was confirmed by performing parallel experiments with control slides and using identical conditions but omitting primary antibodies. Staining with rat anti-ki-67 (Abcam ab156956), mouse anti-CD20, Alexa Fluor 647-rabbit monoclonal anti-CD4 (Abcam ab196147) and rabbit anti-CD3 (Abcam ab16669) was performed similarly on two HLA-DRB1*03^{+} and three HLA-DRB1*03^{-} patient biopsies. However, the primary antibody step involved only rat ki-67, mouse anti-CD20 and rabbit anti-CD3. The secondary antibodies (Molecular Probes, Invitrogen) used were Alexa Fluor 488-donkey anti-rabbit, Alexa Fluor 568-donkey anti-mouse and Alexa Fluor 594-donkey anti-rat. A second blocking step was then performed in a second blocking buffer (TBS/0.02% Triton X-100/10% normal donkey serum/10% normal rabbit serum1:500 Human BD Fc Block [BD Pharmingen]) to saturate free binding sites of the anti-rabbit secondary. The last incubation was performed with Alexa Fluor 647-rabbit monoclonal anti-CD4 diluted in the second blocking buffer (supplemented with Hoechst). Images were acquired using the 40X oil immersion objective on a Leica SP8 Laser Scanning microscope (Leica Microsystems, Wetzlar, Germany) with 1024 x 1024 fields of view. Autofluorescence was subtracted with use of the light-gate module, which together with laser powers and photomultiplier tube settings were kept constant throughout all acquisitions. Raw data files of images for respective channels (inherently grey scaled), corresponding to respective fluorescent emissions and digital image contrast (DIC), were exported as TIF files, assigned false colours (shown in the manuscript), and converted to the RGB image format with Image J freeware (NIH, Bethesda, MD, USA). One round of background subtraction by the automated "despeckling" function was applied and false colours were assigned and colour merging also performed with Image J freeware.

Tiled images were obtained by firstly obtaining individual frames of interest using a 40X objective using gating to subtract background, and autofocusing based on the Hoechst chanel

to account for a 10 micron tissue depth. Tiles, each 235.2μm^2 per field of view overlapped by 10% and were stitched automatically using the "statistical" blend mode in the Leica software. Pixel size of the resulting tiled image was 227.23 ηm^2. Raw grey data from respective colour channels was exported as separated TIF files. TIFs were assigned false colours in Image J and merged into one composite TIF. All channels underwent one round of despeckling noise reduction before merging. Finally, areas of interest ($CD20^+$ clusters) were manually defined using the polygon function on the respective raw grey image (from the channel used for acquiring the CD20 signal), and total areas and pixel intensities within the defined areas measured using the measurement function in Image J.

Peptide digestion and HPLC for mass spectrometry identification of vimentin

Presence of free vimentin in BALF samples (previously removed of cells and particulate matter by centrifugation) was determined through use of samples concentrated through 10MWCO columns (AmiconUltra-15, Millipore, Tullagreen, Ireland) or left unconcentrated before diluting in reducing Laemmli buffer. BALF was resolved by SDS-PAGE and stained with a Coomassie-based reagent (Instant Blue, Expedeon, Cambridge, UK). Proteins were excised for in-gel trypsinisation and mass spectrometry as described previously (S2). Specifically, gel sections in the molecular weight range of 35k-55 kDa were excised by sterile razor blade and digested with trypsin according to previously described methodology (S3). All samples were re-suspended in Burdick & Jackson HPLC-grade water containing 0.2% formic acid (Saint Louis, MO, USA), 0.1% TFA (Thermo Scientific) and 0.002% Zwittergent 3–16 (EMD Biosciences, San Diego, CA), a sulfobetaine detergent that contributes the following distinct peaks at the end of chromatograms: MH^+ at 392, and in-source dimer [$2 M + H^+$] at 783, and some minor impurities of Zwittergent 3–12 seen as MH^+ at 336. The peptide samples were loaded to a 0.25 µl C_8 OptiPak trapping cartridge custom-packed with

Michrom Magic (Optimise Technologies, Oregon City, OR, USA) C8, washed, then switched in-line with a 20 cm by 75 µm C_{18} packed spray-tip nano-column packed with Michrom Magic C18AQ, for a 2-step gradient. Mobile phase A was water/acetonitrile/formic acid (98/2/0.2) and mobile phase B was acetonitrile/isopropanol/water/formic acid (80/10/10/0.2). Using a flow rate of 350 nl/min, a 90 min, 2-step LC gradient was run from 5% B to 50% B in 60 min, followed by 50%–95% B over the next 10 min, hold 10 min at 95% B, back to starting conditions and re-equilibrated.

LC–MS/MS analysis

BALF samples were analysed via electrospray tandem mass spectrometry (LC–MS/MS) on a Q-Exactive Orbitrap mass spectrometer (Thermo Scientific), using a 70,000 RP survey scan in profile mode, m/z 360–2000 Da, with lockmasses, followed by 20 MSMS HCD fragmentation scans at 17,500 resolution on doubly and triply charged precursors. Single-charged ions were excluded, and ions selected for MS/MS were placed on an exclusion list for 60 s.

Statistical analysis of LC–MS/MS data

All LC-MS/MS *.raw data files were analysed with MaxQuant version 1.5.2.8 (Max Planck Institute of Biochemistry, Berlin, Germany) searching against the SPROT human database (downloaded: 3/31/2017 with isoforms) using the following criteria: Label Free Quantitation (LFQ) was selected for quantitation with a min of 1 high-confidence peptide to assign LFQ intensities. Trypsin was selected as the protease with maximum miss-cleavage set to 2. Carbamiodomethyl (C) was selected as a fixed modification. Variable modifications were set to Oxidization (M), Formylation (N-term), Deamidation (NQ), Deamidation (R). Orbitrap mass spectrometer was selected using an MS error of 20 ppm and a MS/MS error of 0.5 Da. A

1% FDR cut-off was selected for peptide, protein, and site identifications. Protein abundance was determined via LFQ intensities of protein peak areas determined by MaxQuant and reported in proteinGroups.txt.

Vimentin cloning and protein purification

Full-length vimentin cloned into the C-terminal 6 His-tag fusion expression vector pET-24b was kindly provided by Mor-Vaknin *et al* (University of Michigan, MI, USA). Three vimentin truncations, each containing the C-terminal 6 His-tag were generated by PCR amplification and cloning back within the BamH1 and Hind111 restriction sites (italicised in primers listed) of the multiple cloning site of the pET-24b vector as detailed in Kinloch *et al* (in preparation), and based on the truncations previously cloned by Cha *et al* (S4). Start and stop codons were incorporated into primers as deemed appropriate (bold). "N-terminal short", corresponding to amino acid sequence 1-137, was amplified from the full-length vimentin vector using primers "fwd vim" (*AAGGATCC*ATGTCCACCAGGTCCGTGTCCTCG) and "Vim137 rev" (AA*AAGCTT*GT**TTA**CTGCTCGAGCTCGGCCAGCAGGATCTT). "N-terminal long", corresponding to amino acid sequence 1-259, was amplified using the primers "fwd vim" and "Vim 259 rev" (AA*AAGCTT*GT**TTA**CACATCGATTTGGACATGCTGTTC). "C-terminal", corresponding to amino acid sequence 260-467, was amplified using primer "Vim 260 fwd" (AA*GGATCC*G**ATG** TTTCCAAGCCTGACCTCACGGCTG) and "Vim C-term rev" (AA*AAGCTT*GT**TTA**TTCAAGGTCATCGTGATG). Expression was induced in BL21 Rosetta (DE3) pLysS (EMD, Madison, WI, USA) with 0.1M IPTG overnight. Cultures were pelleted and subjected to 3 rounds of lysis with lysis buffer (20mMTris-HCl pH 7.5, 0.2M NaCl, 1% NP-40, 10mM NaF, 2mM EDTA) and washed for two rounds with Triton-buffer (10mMTris-HCl pH 7.5, 0.5% Triton, 0.1M NaCl). Intermediate steps of centrifugation, each

at 15000 rpm for 15 min, were performed at room temperature. The inclusion body was then lightly stirred (in 8M urea 50mM Tris-HClpH 8.0 10mM beta-mercaptoethanol) for one hour at room temperature and centrifuged at 15000 rpm. The supernatant was incubated with Ni-agarose resin overnight at 4°C and then washed stepwise with 8M urea with decreasing acidity from pH8.0 to pH3.2. Eluted washes were collected and fractions containing pure (as determined by Coomassie staining) full-length vimentin or vimentin truncations were pooled and dialysed using Snakeskin Dialysis tubing, 10K MWCO (Thermo Scientific, Rockford, IL, USA), against 0.1M Tris-HCl pH7.4, concentrated with 10kDa molecular weight exclusion columns (Amicon, Co. Cork, Ireland) and stored at -80°C.

ELISAs

Anti-vimentin antibody ELISAs were performed as for Kinloch *et al* (in preparation). Briefly, antigen aliquots were solubilised to 1.0mg/ml (in 10% DMSO) and subsequently diluted into PBS for coating ELISA plate (Costar 96-well clear, flat-bottom, half-area high-binding polystyrene plates, Corning cat.no 3690, New York, USA) wells at 10µg/ml. Wells were washed with ddH_20 and blocked for 3 hours with blocking buffer (PBS/3% BSA). Serum or BALF was diluted into blocking buffer and incubated in the well for 1.5 hours and washed five times (one minute per wash) with PBS/Tween 0.1%. Bound IgG or IgA was detected using HRP-conjugated secondary antibodies (109-035-098 and 109-035-011, Jackson ImmunoResearch, West Grove, PA, USA) diluted in blocking buffer, and following a second round of washing, optical density of wells containing Super AquaBlue (eBioscience, San Diego, CA, USA) were measured at 405 nm. For each antigen and isotype, a standard curve was generated by performing two-fold dilutions of frozen aliquots of standards consisting of pooled sera containing high titres of antibody to the respective whole antigen or truncation.

Arbitrary units (AU) of antibody titres for each analyte were interpolated from a standard curve, itself generated by a serial dilution of the respective ELISA's standard (assigned a value of 1000 AU) and subsequently multiplying by its dilution factor. ELISAs were repeated until values could be interpolated within the range of the standard curve. Antibodies titrated to the full-length vimentin molecule (amino acids 1-467) are referred to as anti-vimentin antibodies (AVAs), to the N-terminal truncation (amino acids 1-259) as anti-Vim$_{\text{N-termlong}}$, to the N-terminal truncation (amino acids 1-137) as anti-Vim$_{\text{N-termshort}}$ and to the C-terminal truncation (amino acids 260-467) as anti-Vim$_{\text{C-term.}}$ Neat titres (AU) in the text refer to the interpolated titres multiplied by their respective dilution factors. Concentrations (AU/mg) were calculated by dividing the neat titres (AU) by the analytes' total immunoglobulin isotype concentration.

Total IgG and IgA concentrations in serum and BALF were titrated using Human IgA and Human IgG ELISA kits (E-80A and E-80G, ICL Inc, Portland, OR, USA).

Analyses comparing the difference between groups in terms of relative concentrations of antibodies to the C-terminus and N-terminus were performed by dividing the concentration (AU/mg) of antibodies to the vimentin C-terminus by the concentration (AU/mg) of antibodies to the respective N-terminal fraction for each respective analyte.

frontiers

References

S1. Olerup O, Zetterquist H. HLA-DR typing by PCR amplification with sequence-specific primers (PCR-SSP) in 2 hours: an alternative to serological DR typing in clinical practice including donor-recipient matching in cadaveric transplantation. Tissue Antigens. 1992;39(5):225-35.

S2. Kinloch AJ, Chang A, Ko K, Henry Dunand CJ, Henderson S, Maienschein-Cline M, et al. Vimentin is a dominant target of in situ humoral immunity in human lupus tubulointerstitial nephritis. Arthritis Rheumatol. 2014;66(12):3359-70.

S3. Truman AW, Kristjansdottir K, Wolfgeher D, Hasin N, Polier S, Zhang H, et al. CDK-dependent Hsp70 Phosphorylation controls G1 cyclin abundance and cell-cycle progression. Cell. 2012;151(6):1308-18.

S4. Cha SC, Qin H, Kannan S, Rawal S, Watkins LS, Baio FE, et al. Nonstereotyped lymphoma B cell receptors recognize vimentin as a shared autoantigen. J Immunol. 2013;190(9):4887-98.

Supplementary Figures

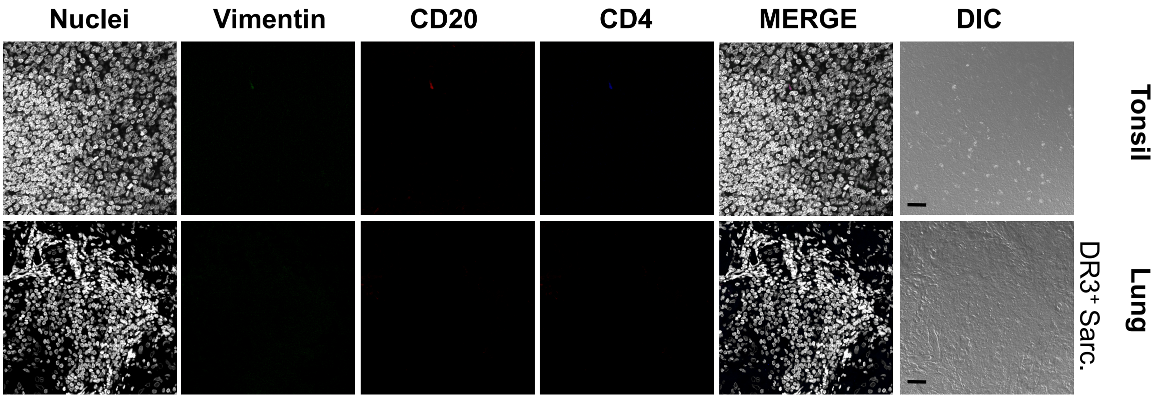

Figure S1. Negative control stains of tonsil and inflamed lung tissue.

Control slides covered with tonsil and highly inflamed HLA-DRB1*03^{+} sarcoid lung tissue (patient matched to those used for Fig. 1) were stained as for Fig. 1, with the omission of the primary antibodies. The absence of fluorescence in the respective channels (excluding the one acquiring Hoechst staining of nuclei) confirmed the signals in the respective channels were neither due to autofluorescence, nor to secondary antibodies directly binding to the tissue on the slide. Images were acquired with an oil immersion 40X objective. Black bar = 25 microns.

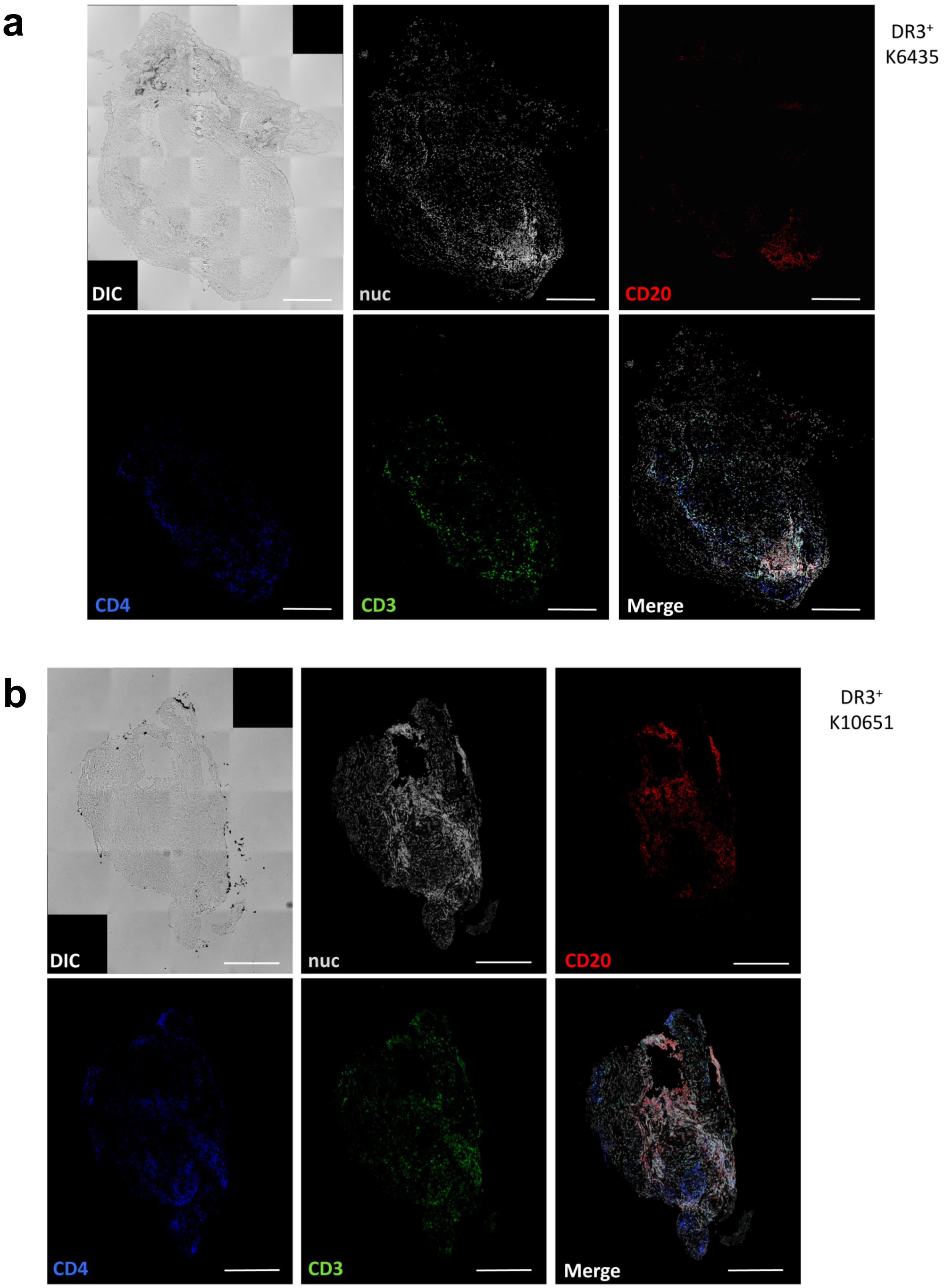
a
DIC
nuc
CD20
CD4
CD3
Merge
DR3+
K6435
b
DIC
nuc
CD20
CD4
CD3
Merge
DR3+
K10651

DR3+ K8660

c

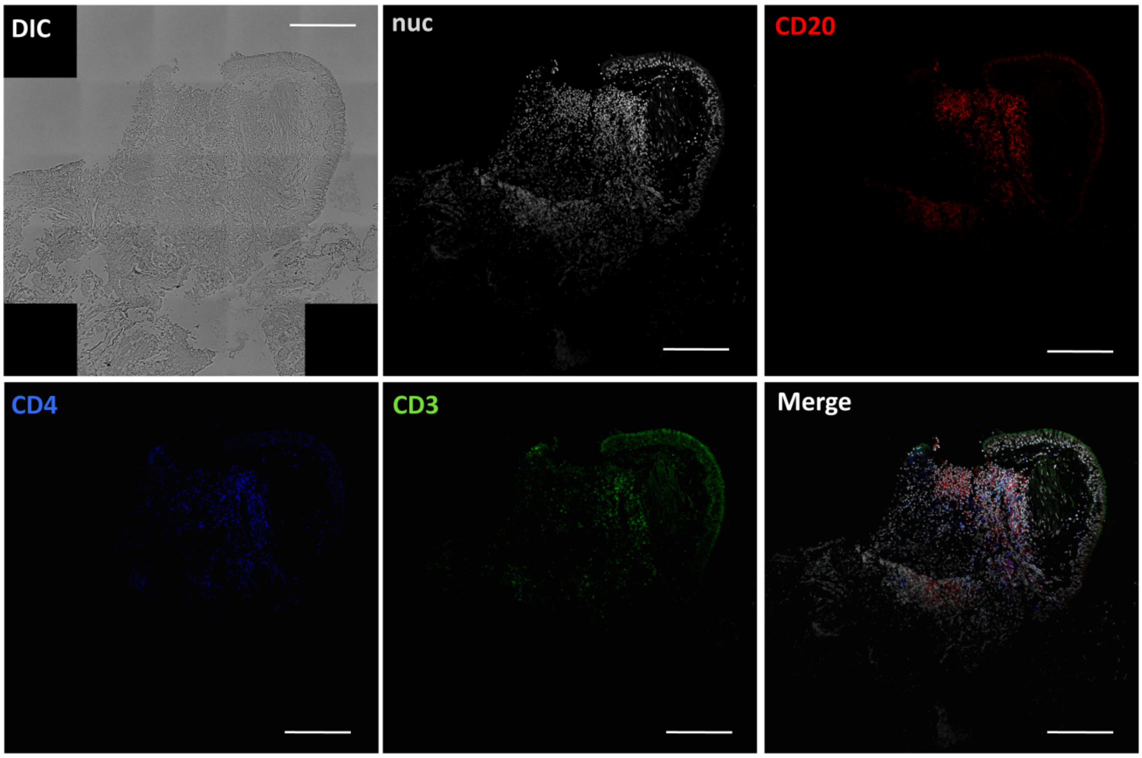

d

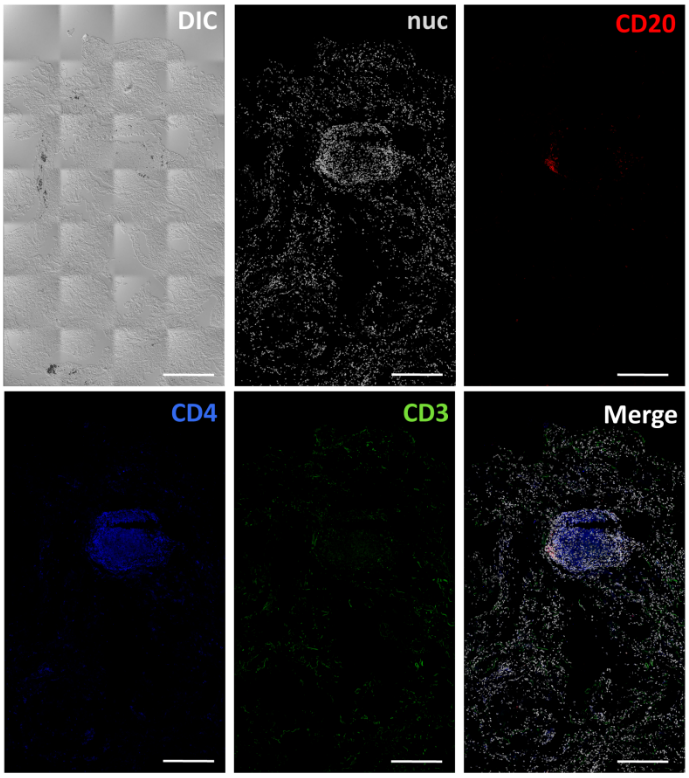

DR3- K15761

e

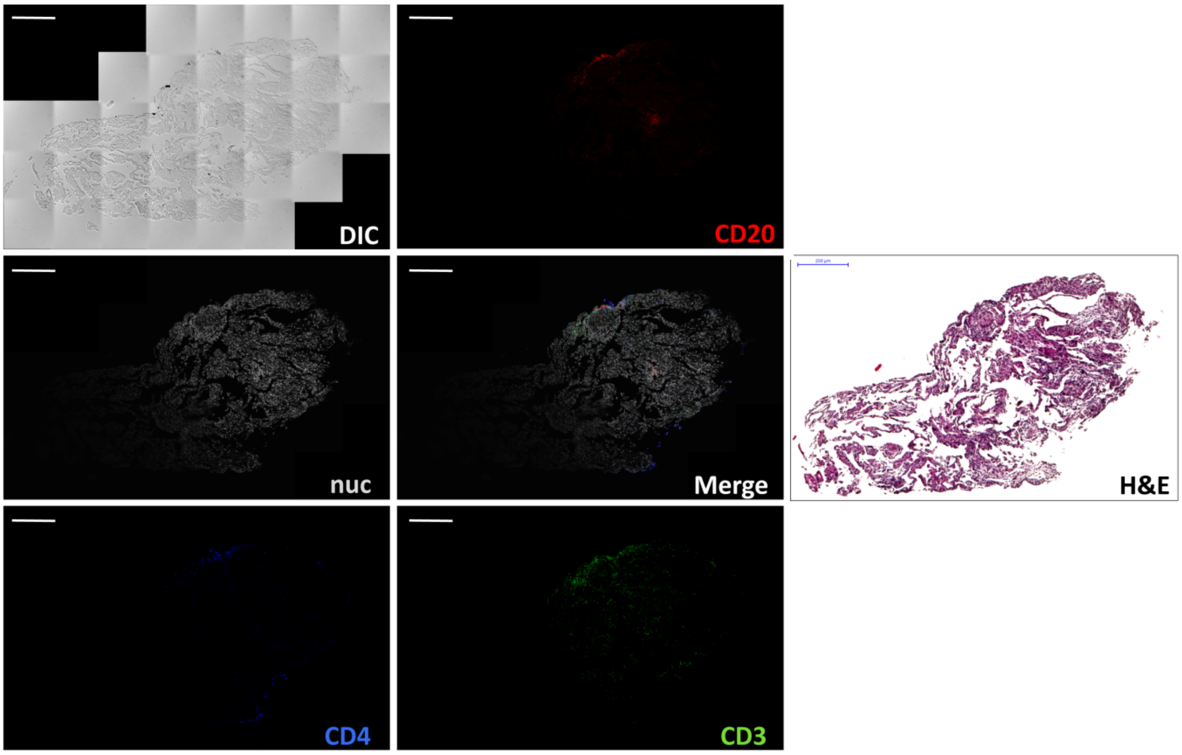

DR3^{-} K4322

f

DR3- K14231

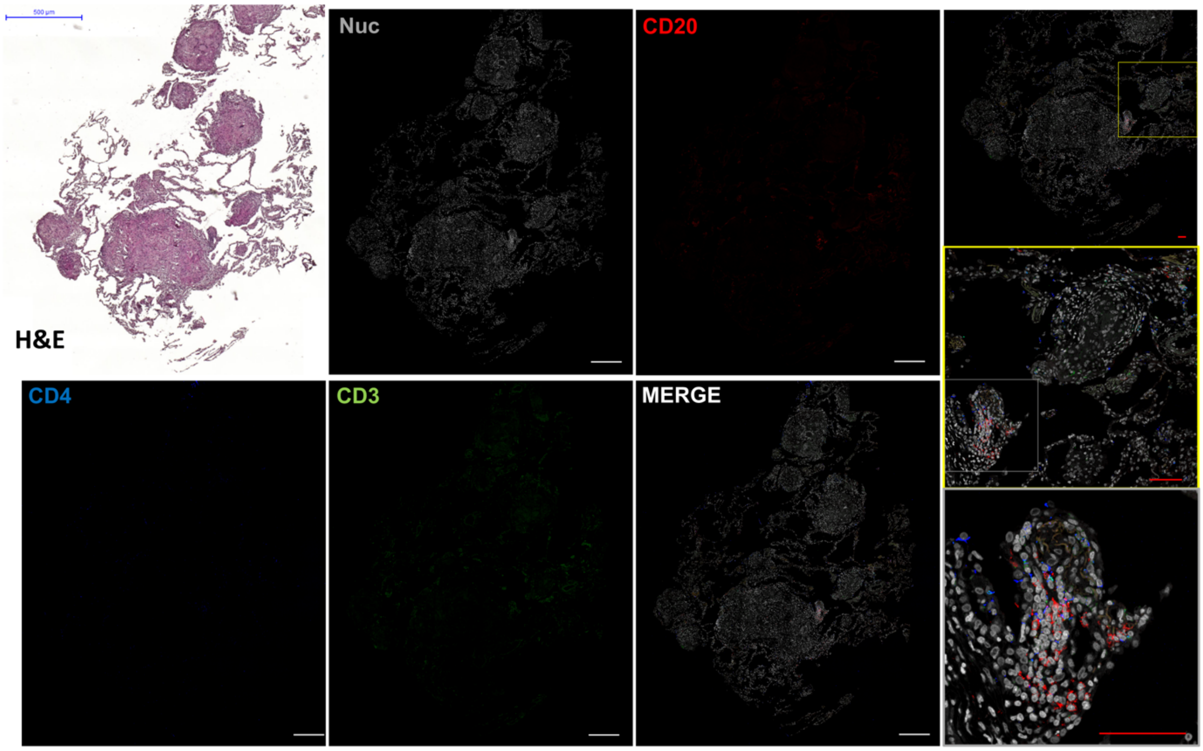

g

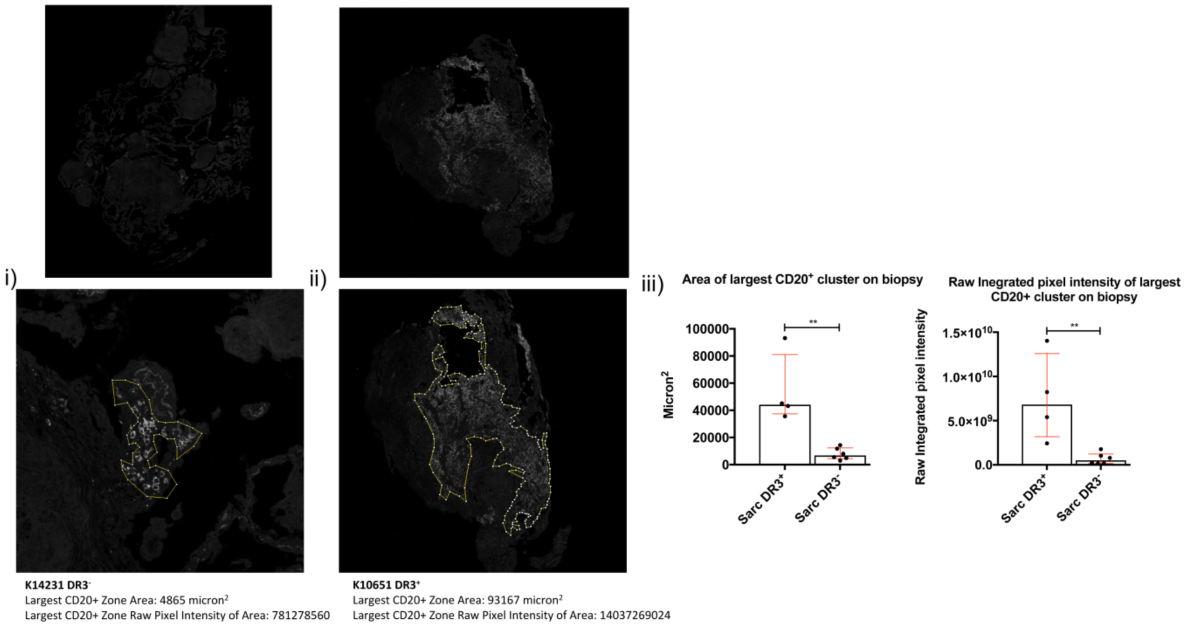

Figure S2. Wide view confocal images of inflamed lung tissue in HLA-DRB1*03 positive and negative patients.

Confocal images (digital stitchings of multiple fields of view) of sarcoid lung tissue from HLA-DRB1*03^{+} **(a-c)** and HLA-DRB1*03^{-} **(d-f)** patients, respectively, obtained using a 40X objective and 10% overlapping 235.2µm^{2} tiles per field of view. Where sufficient numbers of slides from the same patient were available, complementary haematoxylin and eosin (H&E) stains were performed to further visualise the granulomatous structures **(e-f)**. To appreciate the relatively small area of HLA-DRB1*03^{-} lung tissue containing CD20^{+} B-cells, and where these cells locate to, **(f)** provides a series of increasing magnifications of the largest CD20^{+} cell cluster for the patient biopsy in question (white scale bars = 200 microns; red scale bars = 60 microns).

Representative images derived from the CD20^{+} cell channel (546 nm) used for quantification of the largest CD20^{+} cluster in each respective sarcoid lung biopsy are shown in **(g)** for one HLA-DRB1*03^{-} (i, upper panel) and one HLA-DRB1*03^{+} patient (ii, upper panel). Lower panels (i and ii) represent digital magnifications of the largest single CD20^{+} cluster within

each respective biopsy, which was manually defined (yellow border) using the polygon function in the Image J software. The area and raw integrated pixel intensity metrics for the largest $CD20^+$ B-cell cluster for each biopsy were compared between HLA-DRB1*03 positive and negative patients in (iii), showing significantly larger $CD20^+$ B-cell clusters in HLA-DRB1*03^+ patient lung biopsies.

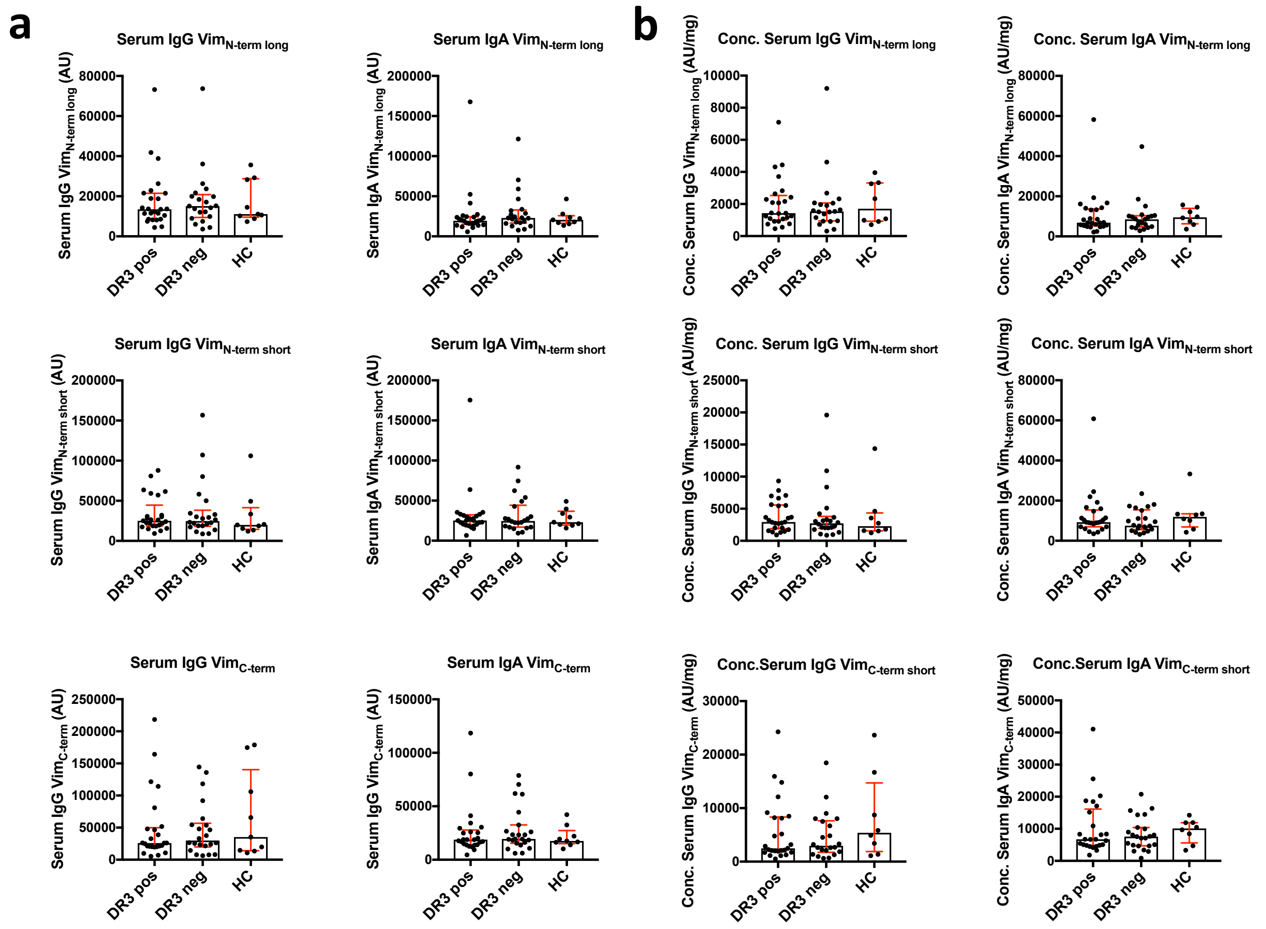

Figure S3. Serum IgG and IgA titres to N- and C-terminal vimentin.

As for Fig. 4, differences in serum titres of IgG and IgA to long ($\mathrm{Vim}_{\text{N-term long}}$) and short N-terminal ($\mathrm{Vim}_{\text{N-term short}}$), and a C-terminal recombinant fraction ($\mathrm{Vim}_{\text{C-term}}$) of vimentin, between sarcoid patient groups and healthy controls (HC) are given as neat values (AU, **(a)**) and as concentrations of total immunoglobulin isotype (AU/mg, **(b)**). Titre comparisons between the two patient groups and HC (healthy controls) were performed using the two-tailed Mann-Whitney U test. No statistically significant differences (p<0.05) were observed between any of the groups for the titrated AVA subtypes.

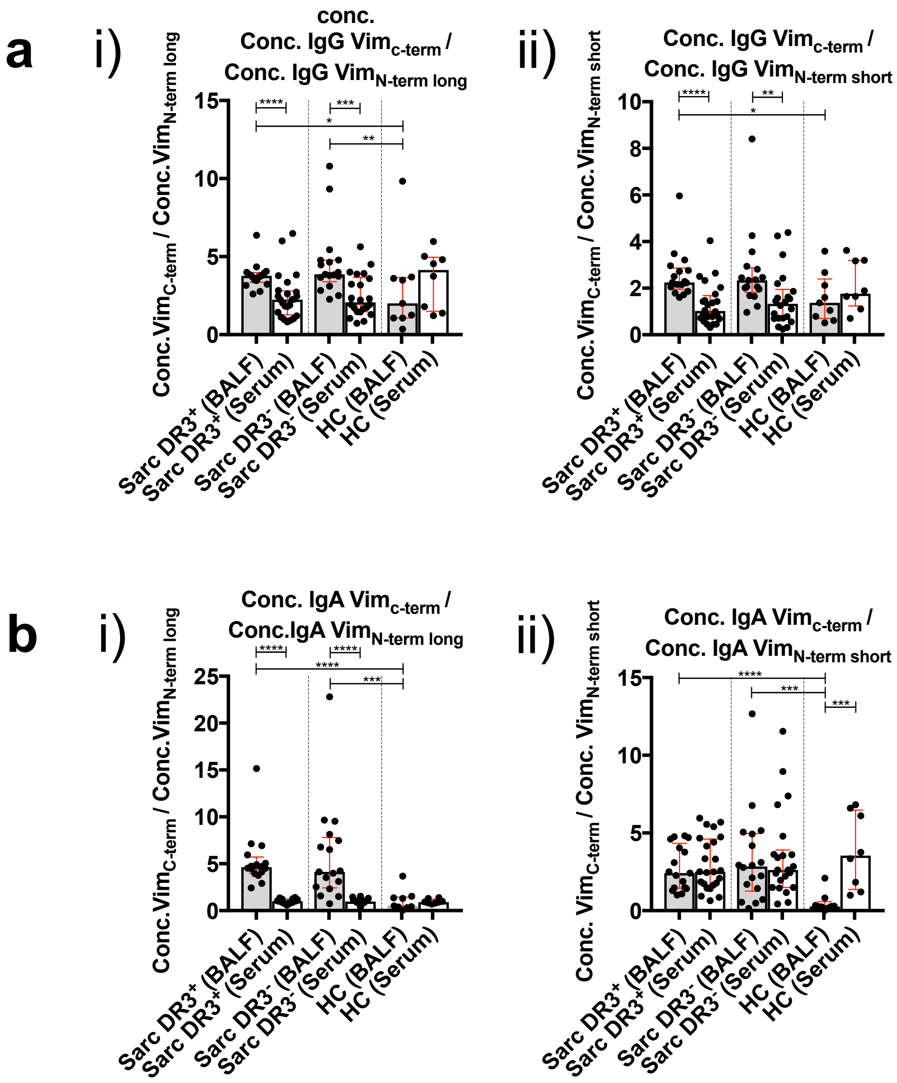

Figure S4. Ratio of C-terminal to N-terminal AVAs in BALF and serum.

Ratios between concentrations (AU/mg) of antibodies to the vimentin C-terminus ($Vim_{C\text{-term}}$) and N-terminus ($Vim_{N\text{-term long}}$ and $Vim_{N\text{-term short}}$) in BALF and serum. For BALF IgG **(a**, i-ii**)** as well as BALF IgA **(b**, i-ii**)**, patients showed significantly more C-terminal-focused reactivity than healthy controls (HC). By this metric, patients who were positive for *HLA-DRB1*03* most frequently (3/4 of the respective ratios) showed the greatest statistical difference from HCs. The sarcoid lung, irrespective of *HLA-DRB1*03* carriage, showed significantly more C-terminal-focused reactivity than the serum (3/4 of the respective ratios), where the healthy lung predominantly (3/4 of the respective ratios) showed statistically insignificant differences with the serum. Comparisons between patient groups and HC were

performed using the non-parametric Mann-Whitney U test. Each dot represents the ratio for the respective BALF or serum sample. Values are presented as median with interquartile range and statistical significances are denoted as two-tailed p-values as follows: *$p<0.05$; ** $p<0.01$; *** $p<0.001$; **** $p<0.0001$

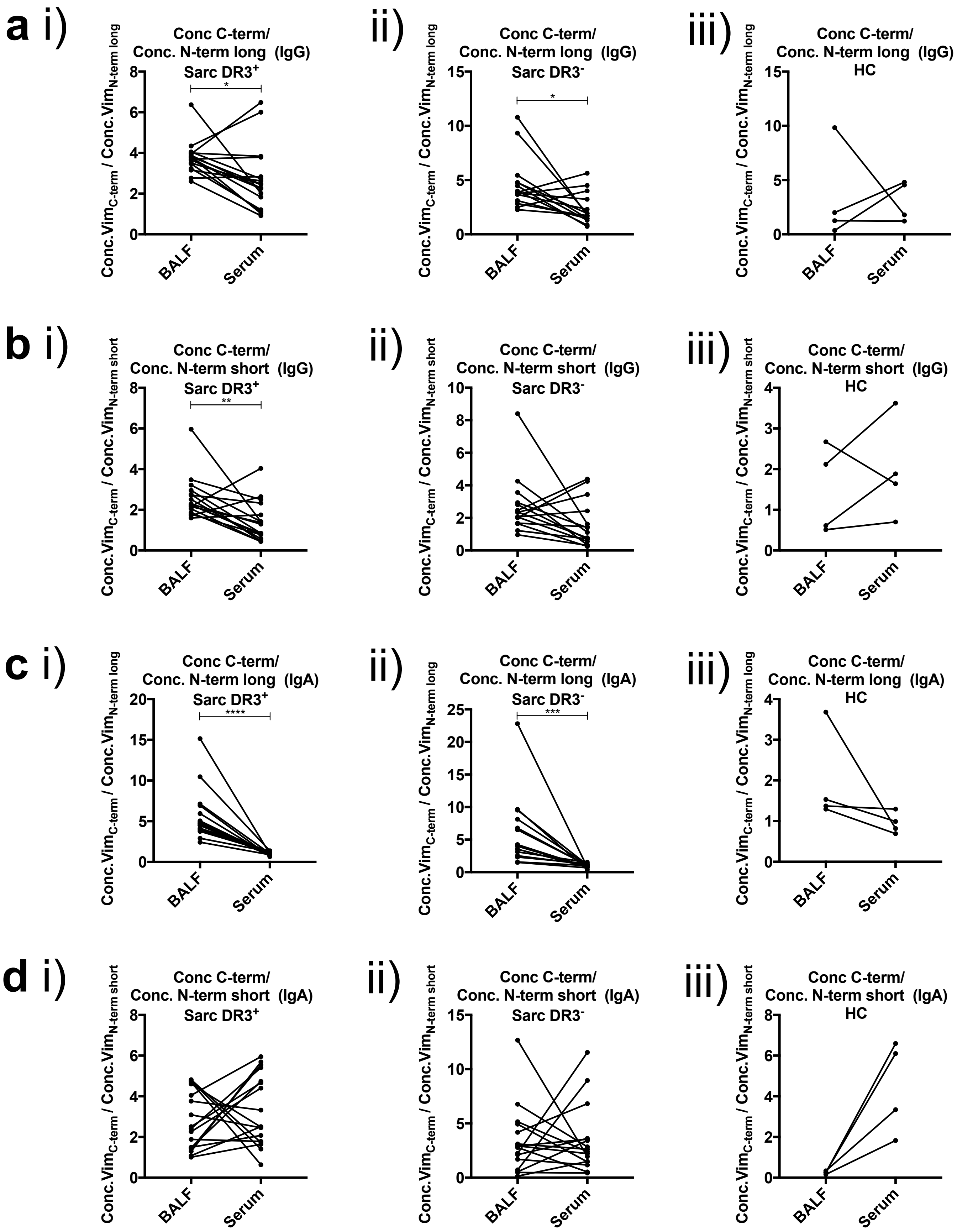

a i)
Conc C-term/
Conc. N-term long (IgG)
Sarc DR3+
*
Conc.VimC-term / Conc.VimN-term long
BALF
Serum
ii)
Conc C-term/
Conc. N-term long (IgG)
Sarc DR3-
*
iii)
Conc C-term/
Conc. N-term long (IgG)
HC
b i)
Conc C-term/
Conc. N-term short (IgG)
Sarc DR3+
**
Conc.VimC-term / Conc.VimN-term short
ii)
Conc C-term/
Conc. N-term short (IgG)
Sarc DR3-
iii)
Conc C-term/
Conc. N-term short (IgG)
HC
c i)
Conc C-term/
Conc. N-term long (IgA)
Sarc DR3+
****
ii)
Conc C-term/
Conc. N-term long (IgA)
Sarc DR3-
***
iii)
Conc C-term/
Conc. N-term long (IgA)
HC
d i)
Conc C-term/
Conc. N-term short (IgA)
Sarc DR3+
ii)
Conc C-term/
Conc. N-term short (IgA)
Sarc DR3-
iii)
Conc C-term/
Conc. N-term short (IgA)
HC

Figure S5. Paired analyses of C-terminal-to-N-terminal AVA ratio in BALF and serum of individual patients and healthy controls.

Ratios between concentrations (AU/mg) of antibodies to the vimentin C-terminus ($Vim_{C\text{-term}}$) and N-terminus ($Vim_{N\text{-term long}}$ and $Vim_{N\text{-term short}}$) in BALF and serum, as presented in Fig. S4, were analysed by pairwise comparisons for individual subjects in the three respective groups: HLA-DRB1*03 positive (**a-d**, i) and negative (**a-d**, ii) sarcoidosis patients, as well as healthy controls (**a-d**, iii). Paired analyses further support the phenomenon demonstrated in Fig. 6 and Fig. S4, with C-terminal reactivity being predominant in the sarcoid, and especially the HLA-DRB1*03^{+}, lung compared to serum. Notably, paired analyses could not be performed for subjects for whom either BALF or serum AVA and/or total Ig data were lacking, thereby reducing statistical power, particularly for the healthy control group where material is more scarce and larger sample volumes are required for analysis due to inherently low Ig titres. Pairwise comparisons were performed using the non-parametric Wilcoxon's signed rank test. Each dot represents the ratio for the respective BALF or serum sample. Statistical significances are denoted as two-tailed p-values as follows: *p<0.05; ** p<0.01; *** p<0.001; **** p<0.0001

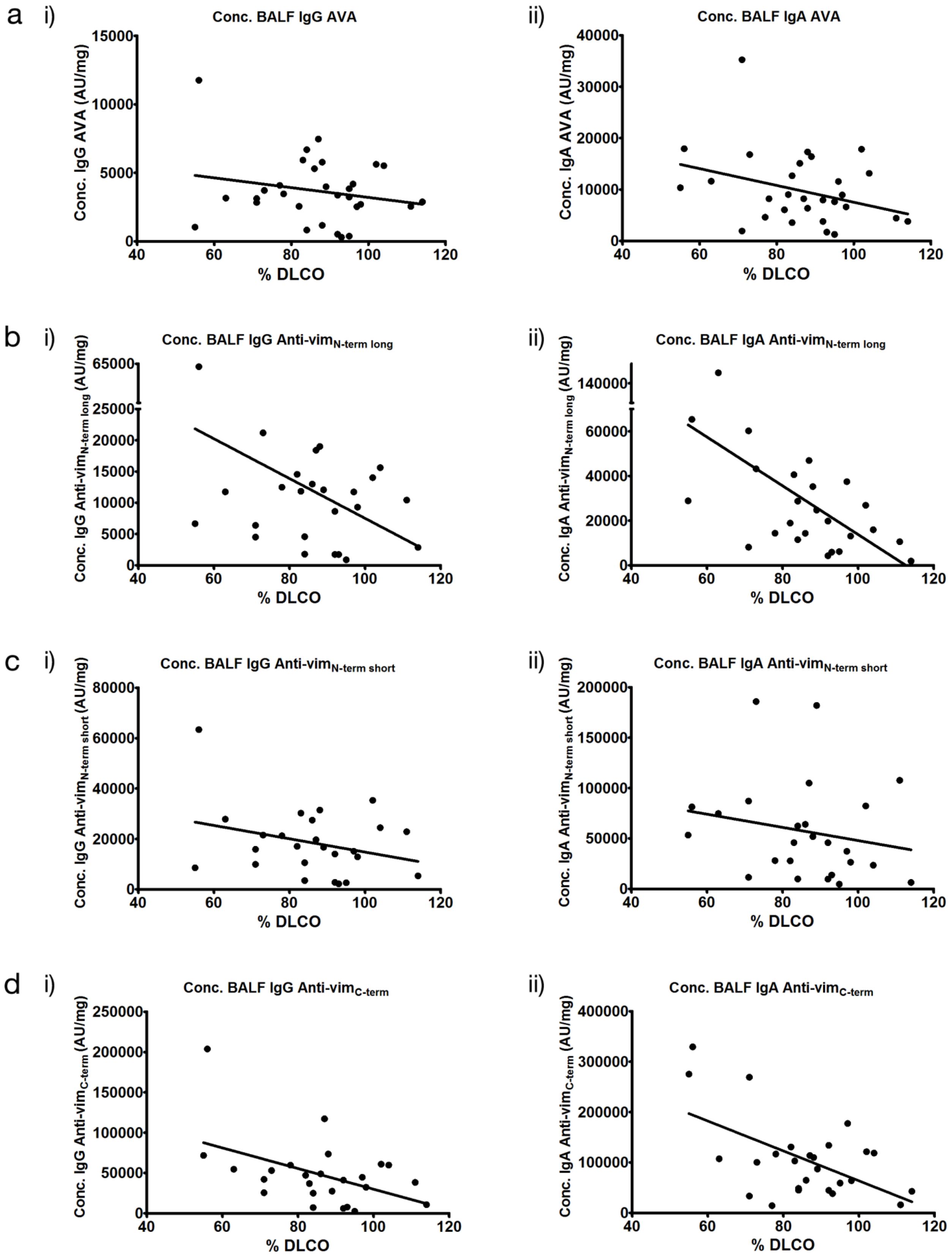
a i)
Conc. BALF IgG AVA
Conc. IgG AVA (AU/mg)
15000
10000
5000
0
40
60
80
100
120
% DLCO
ii)
Conc. BALF IgA AVA
Conc. IgA AVA (AU/mg)
40000
30000
20000
10000
0
% DLCO
b i)
Conc. BALF IgG Anti-vim$_{N\text{-term long}}$
Conc. IgG Anti-vim$_{N\text{-term long}}$ (AU/mg)
65000
25000
20000
15000
10000
5000
0
% DLCO
ii)
Conc. BALF IgA Anti-vim$_{N\text{-term long}}$
Conc. IgA Anti-vim$_{N\text{-term long}}$ (AU/mg)
140000
60000
40000
20000
0
% DLCO
c i)
Conc. BALF IgG Anti-vim$_{N\text{-term short}}$
Conc. IgG Anti-vim$_{N\text{-term short}}$ (AU/mg)
80000
60000
40000
20000
0
% DLCO
ii)
Conc. BALF IgA Anti-vim$_{N\text{-term short}}$
Conc. IgA Anti-vim$_{N\text{-term short}}$ (AU/mg)
200000
150000
100000
50000
0
% DLCO
d i)
Conc. BALF IgG Anti-vim$_{C\text{-term}}$
Conc. IgG Anti-vim$_{C\text{-term}}$ (AU/mg)
250000
200000
150000
100000
50000
0
% DLCO
ii)
Conc. BALF IgA Anti-vim$_{C\text{-term}}$
Conc. IgA Anti-vim$_{C\text{-term}}$ (AU/mg)
400000
300000
200000
100000
0
% DLCO

Figure S6. Correlation of %DLCO with AVA titres in BALF.

Spearman's correlations between diffusion capacity of carbon monoxide (DLCO), a measure of lung function, and BALF IgG and IgA concentrations (AU/mg) to full-length (AVA) **(a)** and truncated vimentin **(b-d)** in HLA-DRB1*03^{+} and HLA-DRB1*03^{-} patients combined. Lines of best-fit are shown, and statistically significant differences are denoted as two-tailed p-values as follows * p <0.05; ** p <0.01. **(a)** i) r=-0.1229, 95% confidence interval -0.4715 to 0.2589, p=0.5176; ii) r=-0.2690, 95% confidence interval -0.5812 to 0.1122, p=1506. **(b)** i) r=-0.1916, 95% confidence interval -0.5542 to 0.2330, p=0.3588; ii) r=-0.548, 95% confidence interval -0.7802 to -01831, p=0.0046**. **(c)** i) r=-0.167, 95% confidence interval -0.5363 to 0.2559, p=0.4249; ii) r=-0.2852, 95% confidence interval -0.6191 to 0.1362, p=0.1671. **(d)** i) r=-0.3244, 95% confidence interval -0.6451 to 0.09348, p=0.1136; ii) r=-0.2781, 95% confidence interval -0.6085 to 0.1344, p=0.1690.

Additional Supporting Files

https://ndownloader.figshare.com/files/12259640

Patient Information File:

"Patient data (deidentified) Kinloch & Kaiser.xslx" (Excel file containing clinical and other metric data on patients and healthy controls used in the study)

Mass Spectrometry files:

"Kinloch Kaiser MaxQuant ProteinGroups.xslx" (Excel file summarising data from BALF samples used for mass spectrometry highlighting identified proteins and vimentin peptides, and their relative abundances)

"Unconcentrated sample gel.Sf3" (Scaffold file demonstrating vimentin peptides and post-translational modifications identified from neat BALF samples boiled in reducing Laemmli buffer, as for Fig.3b [top gel])

"Concentrated sample gel.Sf3" (Scaffold file demonstrating vimentin peptides and post-translational modifications identified from concentrated BALF samples boiled in reducing Laemmli buffer, as for Fig.3b [bottom gel])

to view Scaffold files, Scaffold Viewer freeware requires uploading from the following site: http://www.proteomesoftware.com/products/scaffold/download/
